# Evolutionary origins of ultrasonic hearing and laryngeal echolocation in bats inferred from morphological analyses of the inner ear

**DOI:** 10.1186/1742-9994-10-2

**Published:** 2013-01-30

**Authors:** Kalina TJ Davies, Ibnu Maryanto, Stephen J Rossiter

**Affiliations:** 1 School of Biological & Chemical Sciences, Queen Mary University of London, Mile End Road, E1 4NS, London, United Kingdom; 2 Bogor Zoological Museum, LIPI, Jl. Raya Jakarta-Bogor, KM. 46, Cibinong, Bogor, Indonesia

**Keywords:** Cochlea, Echolocation, Traits, Ancestral reconstruction, Hearing

## Abstract

**Introduction:**

Many mammals have evolved highly adapted hearing associated with ecological specialisation. Of these, bats possess the widest frequency range of vocalisations and associated hearing sensitivities, with frequencies of above 200 kHz in some lineages that use laryngeal echolocation. High frequency hearing in bats appears to have evolved via structural modifications of the inner ear, however, studying these minute features presents considerable challenges and hitherto few such attempts have been made. To understand these adaptations more fully, as well as gain insights into the evolutionary origins of ultrasonic hearing and echolocation in bats, we undertook micro-computed tomography (μCT) scans of the cochleae of representative bat species from 16 families, encompassing their broad range of ecological diversity. To characterise cochlear gross morphology, we measured the relative basilar membrane length and number of turns, and compared these values between echolocating and non-echolocating bats, as well as other mammals.

**Results:**

We found that hearing and echolocation call frequencies in bats correlated with both measures of cochlear morphology. In particular, relative basilar membrane length was typically longer in echolocating species, and also correlated positively with the number of cochlear turns. Ancestral reconstructions of these parameters suggested that the common ancestor of all extant bats was probably capable of ultrasonic hearing; however, we also found evidence of a significant decrease in the rate of morphological evolution of the basilar membrane in multiple ancestral branches within the Yangochiroptera suborder. Within the echolocating Yinpterochiroptera, there was some evidence of an increase in the rate of basilar membrane evolution in some tips of the tree, possibly associated with reported shifts in call frequency associated with recent speciation events.

**Conclusions:**

The two main groups of echolocating bat were found to display highly variable inner ear morphologies. Ancestral reconstructions and rate shift analyses of ear morphology point to a complex evolutionary history, with the former supporting ultrasonic hearing in the common bat ancestor but the latter suggesting that morphological changes associated with echolocation might have occurred later. These findings are consistent with theories that sophisticated laryngeal echolocation, as seen in modern lineages, evolved following the divergence of the two main suborders.

## Introduction

The evolutionary success of mammals can in part be attributed to remarkable sensory diversification e.g. [1]. While some lineages show evidence of changes across multiple sensory modalities e.g. [2] others have evolved one highly specialised system e.g. [3]. Mammalian auditory systems are particularly well-developed compared to those of many other vertebrate groups, furthermore, mammalian hearing can be characterised by high sensitivity and selectivity and in particular by broad frequency ranges with high upper frequency limits [4,5]. Multidisciplinary evidence suggests that these auditory adaptations in mammals e.g. [6,7] can be linked to three principal adaptations: the evolution of three ossicles in the middle ears (malleus, incus and stapes), elongation of the basilar membrane in the cochlea which provides a supportive base for the sensory hair cells and the evolutionary innovation of outer hair cells (OHC) [8-10]. However, genetic studies suggest additional molecular changes have occurred in the motor protein of the OHC, known as *Prestin*[11], that are probably related to the acquisition of high frequency hearing seen in extant therian mammals [12]. Therefore, it has recently been summarized that true high frequency hearing (i.e. >20 kHz) likely evolved approximately 125 million years (Ma) ago within the therian lineage and required additional structural modifications of the inner ear (as reviewed in [13]).

The mammalian cochlea is a coiled cavity in which high and low frequency sounds are perceived by the basal and upper turns respectively, and this tonotopic organisation is partly achieved by a decrease in basilar membrane stiffness from base to apex [14,15]. Mammals exhibit considerable variation in hearing capabilities and cochlear morphology [16], although some consistent relationships link these two traits. Basilar membrane length is positively correlated with body mass [17], and absolute basilar membrane length is negatively correlated with both high and low frequency hearing limits e.g. [18,19]. It has been hypothesised that cochlear coiling evolved in response to selection pressures relating to the accommodation of elongated auditory sensory membranes of the inner ear [5], however, a previous study found no significant relationship between the number of cochlear turns and basilar membrane length [18]. Recent evidence suggests that coiled cochleae may play a mechanical role in low frequency hearing limit [20]. Across auditory ‘generalists’ high frequency hearing limits correlate with inter-aural distance [21], whereas auditory specialists such as subterranean mole rats and echolocating cetaceans deviate from this relationship [4]. Other factors determining the morphology of auditory systems include physical and mechanical constraints, as well as phylogeny e.g. [16,22,23].

Although several mammalian taxa, including some rodents, carnivores and primates are capable of either detecting or producing ultrasonic sounds (>20 kHz) [4,24] the most highly developed auditory systems for perceiving ultrasonic sound are seen in toothed whales and laryngeal echolocating bats [16,25]. Bats possess some of the widest frequency ranges of vocalisations and, therefore, assumed associated hearing sensitivities of any mammal group, with recorded vocalisations ranging from below 20 kHz to over 200 kHz across the order (as reviewed in [26,27]). Of 19 currently recognised bat families, all but one (the Old World fruit bats) use laryngeal echolocation for orientation, obstacle avoidance and, in most taxa, prey detection [28]. The inner ears of laryngeal echolocating bats show several structural adaptations for detecting ultrasonic echoes; in particular, their cochleae are often enlarged and contain 2.5 to 3.5 turns compared to only an average of 1.75 in non-echolocating fruit bats [29-31]. Furthermore, different forms of echolocation appear to have led to different and sometimes convergent inner ear adaptations [32,33]. For example, both Old World horseshoe bats and the New World moustached bat possess a greatly enlarged cochlear basal turn, which allows exquisite tuning of the inner ear to the echoes of the specialised constant frequency (CF) calls produced by these taxa [34,35]. Previous studies suggest specific adaptations of the anchoring system and the width of the basilar membrane in echolocating bats [36].

Well-supported phylogenies of bats show that laryngeal echolocation is distributed across two highly divergent suborders of bats, termed Yinpterochiroptera and Yangochiroptera, the former of which also contains the non-echolocating Old World fruit bats [37]. To account for this pattern, two main evolutionary scenarios have been proposed; first, that echolocation evolved once in the common ancestor of all modern bats with subsequent loss in Old World fruit bats, and two, that laryngeal echolocation evolved multiple times across the order [38,39]. While fossils bats from the early Eocene have been taxonomically classified as falling outside of the modern bats, and thus might not inform this issue [40,41], recent reports that echolocating members of the two suborders have undergone convergent amino acid replacements in several ‘hearing genes’ would appear to support the multiple origin hypothesis see [42-45].

Evolutionary modifications of the inner ear are likely to have arisen from selection acting on numerous loci, and thus a comparative analysis of morphology might offer a powerful means of reconstructing the origins of ultrasonic hearing and thus laryngeal echolocation in bats. Here we reconstructed three-dimensional bat inner ear volumes of a range of bat species from 16 families, encompassing the broad diversity of echolocation call types and ecological traits seen in the order. We compared patterns of cochlear morphological variation - as defined by relative basilar membrane length and number of turns - among bats, and also between bats and other mammals for which data were available. Correlations were investigated between high and low hearing frequency limits and also echolocation call parameters and morphological characters of the cochlea. We predicted that echolocating bat species would show specific adaptations in aspects of cochlear gross morphology compared to both non-echolocating bats and other mammal species, due to the particular demands associated with receiving the high frequency sounds produced during laryngeal echolocation.

If significant inner ear adaptations for ultrasonic hearing do occur, then these patterns might help us to distinguish between the two most parsimonious alternate scenarios of the evolution of echolocation. For example, it may be possible to determine whether Old World fruit bats show evidence of a loss of echolocation. Alternatively there may be evidence of functional adaptation in the inner ears of the two groups of echolocating bats. Specifically, if Old World fruit bats have lost echolocation, then we might expect to find signatures of this in their cochleae such as intermediate forms between those of echolocating bats and non-echolocating mammals or increased morphological variability, consistent with morphological relaxation.

The two alternate evolutionary scenarios that have been proposed to account for the distribution of laryngeal echolocation across divergent clades of bats might also be expected to have left different traces of inner ear morphological evolution across the bat phylogenetic tree. Firstly, given a single origin in the bat common ancestor we might expect to see increased rates of morphological change in the ancestral bat branch, possibly coupled with a subsequent rate shift in the non-echolocating Old World fruit bats corresponding to a loss of structures associated with sophisticated echolocation capability. In contrast, multiple origins might be expected to leave a signal of morphological rate shifts on specific branches after the point that the two main bat suborders diverged.

## Results

### Cochlear morphology

#### Basilar membrane length

**Figure 1:**
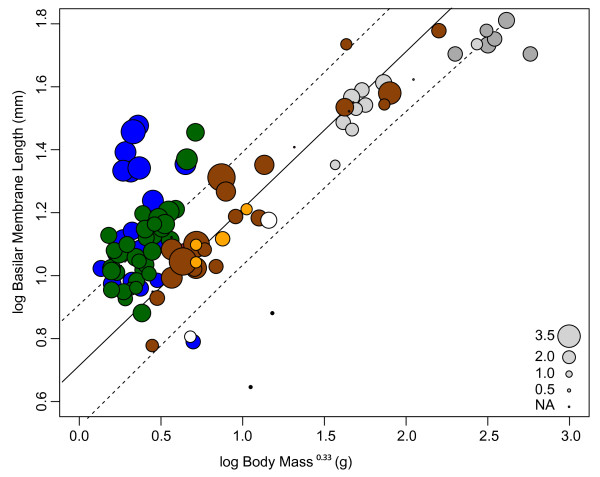
**Log basilar membrane length (mm) versus log body mass**^**0.33 **^**(g) plotted across mammals.** Circles are sized according to the number of turns in the cochlea, and are colour coded as follows: monotremes (black); marsupials (white); non-whale and non-bat placental mammals (brown); echolocating whales (light grey); non-echolocating whales (dark grey); Yangochiroptera (green); echolocating Yinpterochiroptera (blue); Old World fruit bats (orange). The regression line (ordinary least squares) for placental mammals excluding all bats and whales (log basilar membrane = 0.498 log body mass^0.33^ + 0.717, *n* = 25, *R*^2^ = 0.907, F = 225.343, *P* = 2.25 x 10 ^-13^) is shown by the solid line and the 95% prediction intervals for this placental mammal sub-sample is represented by the dashed line. This is similar to the regression results for all non-echolocating mammals (with baleen whales and Old World fruit bats omitted): log basilar membrane = 0.503 log body mass^0.33^ + 0.664, (*n* = 29, *R*^2^ = 0.702, F = 63.877, *P* = 1.37 x 10 ^-8^).

A plot of log basilar membrane length (mm) versus log body mass^0.33^ revealed that a substantial proportion of laryngeal echolocating bats fell above the upper 95% prediction intervals (PI) of the regression line based on non-echolocating placental mammals (excluding baleen whales and Old World fruit bats) (see Figure 1). Most of these outliers were bat species that use constant frequency (CF) echolocation, with the exceptions of *Taphozous peli* and *Cheiromeles torquatus.* Just one laryngeal echolocating bat - *Macroderma gigas* - fell below the lower 95% PI. In contrast to the laryngeal echolocating bats, all non-echolocating Old World fruit bats fell close to the placental mammal regression line and within the PIs. Of the cetacean species plotted, all toothed whales fell within the PI, however, several baleen whales fell outside of the lower 95% PI, and only one placental mammal - the Californian sea lion, *Zalophus californianus -* fell above the upper 95% PI. Of the two marsupial species, one, *Didelphis virginiana*, fell within the placental mammal distribution and the other, *Monodelphis domestica,* just below. Values for the platypus, *Ornithorhynchus anatinus,* and the echidna, *Tachyglossus aculeatus,* clearly fell below the placental mammal prediction interval consistent with them having shorter relative basilar membrane lengths. However, the small sample size of both monotremes and marsupials (*n* = 2 in each case) should be noted.

Bayesian phylogenetic mixed model comparisons revealed no consistent difference in the basilar membrane length of either laryngeal echolocating bats or CF echolocating bats compared to those of other mammals. Across all taxa sampled laryngeal echolocation was not a significant factor (*P*_*MCMC*_ = 0.059) and did not result in improved model fit (ΔDIC = −0.852). Although CF echolocation was a significant factor (*P*_*MCMC*_ = 0.016), it too did not result in an improved model fit (ΔDIC = −0.602). Similar results were obtained in an analysis with a reduced dataset, in which only non-whale placental mammals were included (see supplementary results Additional file 1: Table S5). Baleen and toothed cetaceans were omitted due to their specialised low- and high-frequency hearing respectively, and also due to their much larger body mass.

#### Number of spiral turns

**Figure 2:**
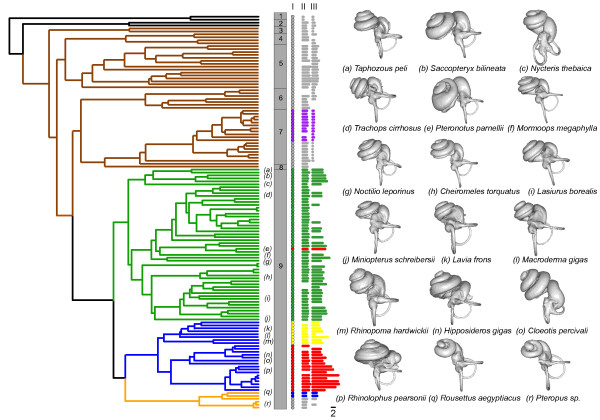
**Phylogeny of the study species with key characters mapped.** Characters are coded as follows: (I) echolocation type: broadband (yellow); constant frequency (red); FM (green); none (grey); tongue-clicks (blue); non-laryngeal echolocation (purple); (II) number of cochlear turns and (III) relative basilar membrane length. Clades are numbered as follows: (1) monotremes; (2) marsupials; (3) Afrotheria; (4) Primates; (5) Glires; (6) Carnivora; (7) Cetartiodactyla; (8) Perissodactyla; (9) Chiroptera. Branch colours indicate: Monotremes and marsupials (black); Non-bat placental mammals (brown); Yangochiroptera (green); echolocating Yinpterochiroptera (blue); non-echolocating Old World fruit bats (Pteropodidae) (orange). Representative examples of cochlear reconstructions for 18 species (***a-r***) are shown.

To test the hypothesis that increased cochlear coiling evolved in response to selection pressures relating to the accommodation of elongated auditory sensory membranes, correlations were investigated between numbers of turns and relative basilar membrane lengths (Additional file 1: Table S2 and Figures 1 and 2). Since cochlear coiling might be predicted to be more important in smaller bodied taxa, the relative membrane length was calculated by dividing membrane length by body mass^0.33^. Across all taxa sampled, a significant positive correlation was found between log number of turns and log relative membrane length (log turns = 0.123 log relative membrane + 0.342, RSE = 0.122 (98 d.f.), *R* = 0.45, adjusted *R*^2^ = 0.19, F = 24.9 (1, 98 d.f., *P* = 2.68 × 10^-6^), which remained significant after accounting for phylogeny (log turns = 0.099 log relative membrane + 0.246, DIC: -321.79, *P*_*MCMC*_(Intercept) < 5 × 10^-4^, *P*_*MCMC*_(log relative membrane) = 0.024).

### Echolocation call analysis

Step-wise multiple regression analyses were used to assess whether echolocation call parameters (i.e. minimum frequency, maximum frequency and peak frequency) relate to both cochlear morphology (basilar membrane length and number of cochlear turns) and body mass across 62 laryngeal echolocating bats and one species of Old World fruit bat that uses tongue-clicking as a rudimentary form of echolocation (*Rousettus aegyptiacus*). After accounting for phylogeny, basilar membrane length and number of cochlear turns both showed significant relationships with all three echolocation call parameters, while body mass did not (Additional file 2: Figure S2 for plots and multiple regression statistics). Furthermore, the two cochlear variables were both significant when fitted together, improving fit (ΔDIC) over univariate models. For minimum echolocation frequency, ΔDIC = 9.48 (Min. frequency = −0.89 log membrane + 2.25 log number of turns + 1.66, DIC: -65.98, *P*_*MCMC*_(log membrane) <1 × 10^-4^, *P*_*MCMC*_(log turns) <1 × 10^-4^. *P*_*MCMC*_(intercept) <1 × 10^-4^). For peak echolocation frequency, ΔDIC = 4.83, (Peak frequency = −0.88 log membrane + 2.00 log number of turns + 1.88, DIC: -53.44, *P*_*MCMC*_(log membrane) <1 × 10^-4^, *P*_*MCMC*_(log turns) < 5 × 10^-4^, *P*_*MCMC*_(intercept) <1 × 10^-4^). For maximum echolocation frequency, ΔDIC = 4.89, (Max. frequency = −0.78 log membrane + 1.44 log number of turns + 2.09, DIC: -50.31, *P*_*MCMC*_(log membrane) = 8.90 × 10^-4^, *P*_*MCMC*_(log turns) = 0.01, *P*_*MCM*C_(intercept) <1 × 10^-4^). Full details are given in Additional file 1: Table S6.

### Ancestral reconstructions of inner ear morphology, hearing and echolocation

**Figure 3:**
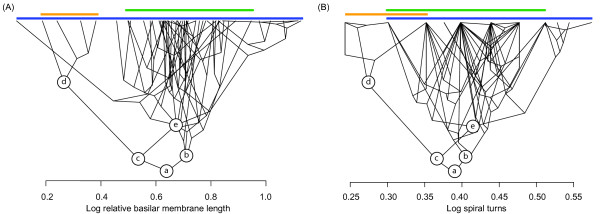
**Ancestral reconstruction of bat (A) relative basilar membrane length and (B) number of cochlear turns using phylogenetic independent contrasts.** Phylogenies and character values are depicted as ‘Traitgrams’, whereby the position along the y-axis corresponds to node age in millions of years and position along the x-axis corresponds to the reconstructed character value. Coloured bars indicate key subdivisions within bats: Old World fruit bats (orange); echolocating Yinpterochiroptera (blue); Yangochiroptera (green). In each traitgram, keys nodes are depicted as follows: bat common ancestor (**a**); Yangochiroptera common ancestor (**b**); Yinpterochiroptera common ancestor (**c**); Old World fruit bat common ancestor (**d**); echolocating Yinpterochiroptera common ancestor (**e**).

**Figure 4:**
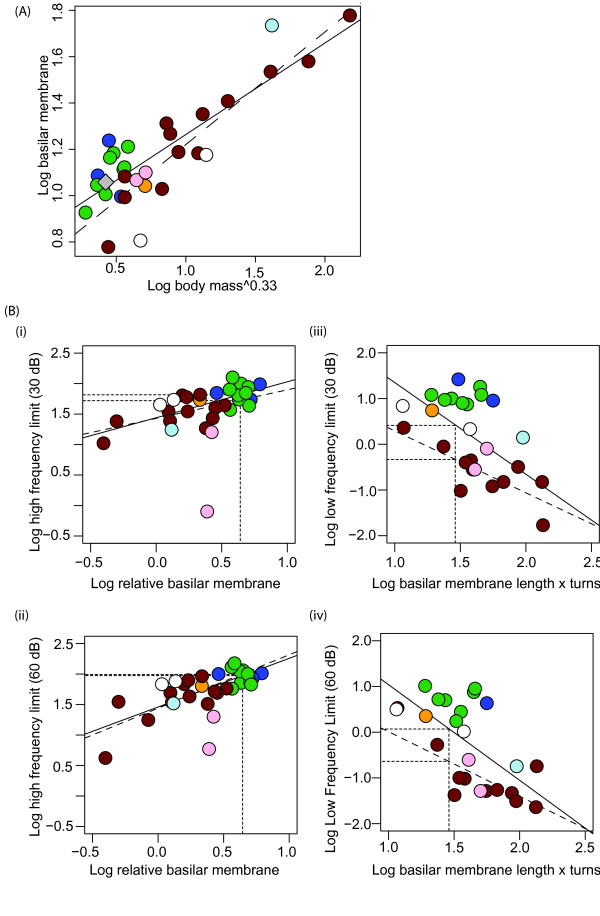
**(A) Relationship between log basilar membrane length and log body mass**^**0.33**^**.** Coloured points indicate the hypothetical modern bat common ancestor bat (grey diamond); Old World fruit bats (orange); echolocating Yinpterochiroptera (blue); Yangochiroptera (green); non-bat placental mammals (brown); subterranean mole-rats (pink); semi-aquatic seals (light blue); marsupials (white). The regression lines are shown for the placental mammal species, including bats (solid line) and excluding bats (dashed line). (**B**) Estimated hearing limits of the hypothetical modern bat common ancestor based on ancestral reconstructions of inner ear morphology. High frequency hearing limits at (i) 30 and (ii) 60 dB, respectively, are estimated using the relationship between log relative membrane length and hearing limits in extant taxa. Low frequency hearing limits at (iii) 30 and (iv) 60 dB, respectively, are estimated using the relationship between the product of log basilar membrane length and the number of cochlear turns with low frequency hearing limits in extant taxa, following [18,20]. For colour coding see Figure 4a.

Phylogenetic independent contrast and maximum likelihood ancestral reconstructions of both log relative membrane length and log number of cochlear turns are shown in Figure 3 and Additional file 3: Figure S3, respectively. Both methods indicated that the log relative basilar membrane length of the hypothetical common ancestor of modern bats was 0.64 mm/g^0.33^, which corresponds to a basilar membrane length of 11.75 mm based on a mass of 19.22 g. Similarly, both methods gave an approximately equal number of cochlear turns: 2.45 (anti-logged). These values suggest a high frequency hearing limit of ~100 kHz at 60 dB and ~60 kHz at 30 dB, with low frequency hearing limit of ~1 kHz at either 30 or 60 dB (see Figure 4).

Based on our ancestral reconstructions of basilar membrane length and cochlear turns, it is not possible to predict whether or not the common ancestral bat was capable of sophisticated laryngeal echolocation. However, under the proposed scenario of a single origin of echolocation, then the observed relationship between inner ear characters and echolocation call frequencies in modern echolocating bats would suggest that the hypothetical call frequencies of the ancestor would have been around ~40 kHz for the minimum call frequency, ~65 kHz for the maximum call frequency, and ~50 kHz for the frequency of peak energy (Additional file 2: Figure S2 for comparison with the echolocating bats included in the study).

### Rate shifts in inner ear morphology

**Figure 5:**
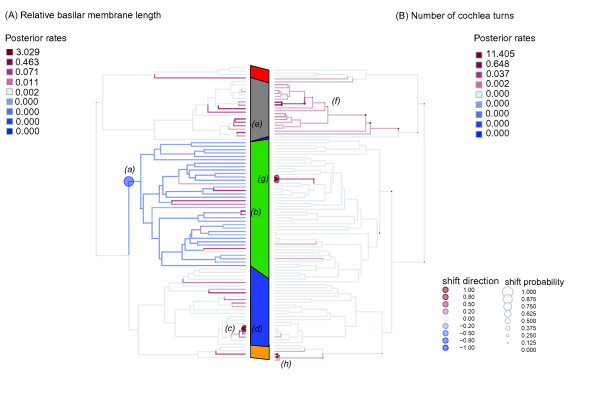
**Evolutionary rate shifts in morphological feature of Laurasiatheria inner ears: (A) log relative basilar membrane length and (B) log number of cochlear turns.** Posterior rates of morphological change in log relative basilar membrane length and log number of turns are indicated by branch colour [decrease in rate (blue) or increase in rate (red), in relation to the background rate (grey)], with the posterior probability of a rate shift occurring at a particular node indicated by the size of the filled circle (see legend for values). Clades are coloured as follows: Carnivora (red); Cetartiodactyla (grey); Perissodactyla (purple); Yangochiroptera (green); echolocating Yinpterochiroptera (blue); Old World fruit bats (orange). Clades of interest that show rate shifts are labelled as follows: (***a***) Yangochiroptera; (***b***) *Molossus spp.*; (***c***) sub-clade of *Rhinolophus* species; (***d***) *Rhinolophus philippinensis* morphs; (***e***) sub-clade of baleen whales; (***f***) Cetartiodactyla; (***g***) *Sturnira spp.*; (**h**) *Pteropus spp* .

An analysis of the rate of morphological change in relative basilar membrane length revealed no change in the rate on the branch leading to all modern bats, as might be expected under the hypothesis of a single gain of echolocation with a subsequent loss in Old World fruit bats. At the same time, most Yangochiroptera branches were shown to have undergone a decrease in rate; this shift was detected with a significant posterior probability (> 0.95) at the basal ancestral Yangochiroptera node (Figure 5A). The only exception to this slowing down was seen in the terminal branches, such as those leading to two *Molossus* species. A much clearer signature of accelerated basilar membrane evolution was seen in the sub-clade of *Rhinolophus philippinensis* morphs, albeit with low probability (see Figure 5A). This latter result is of particular interest as these bats are thought to have undergone recent divergence, putatively linked to a harmonic shift in their echolocation call frequency. Our analysis of echolocation calls of the three *R. philippinensis* morphs suggested that the large morph’s frequency is lower than expected given its forearm size, and the small morph’s is lower than expected given its basilar membrane length (Additional file 4: Figure S4). Apart from within the bats, a sub-clade of baleen whales and California sea lion, *Zalophus californianus*, were also shown to have undergone an increase in the rate of morphological change along their branches.

In contrast to membrane length, rates of change in the number of cochlear turns did not reveal any consistent significant shifts across any bat sub-clade, and instead several increases in rate were detected in scattered clusters of mainly terminal branches (Figure 5B). All Cetartiodactyla branches displayed signatures of accelerated evolution in the number of cochlear turns.

Tests of phylogenetic signal for the three morphological traits showed that the calculated λ value of 0.899 for log basilar membrane length was significantly different from both 0 and 1 (*P* = 1.87 × 10^-28^ and 1.30 × 10^-24^, respectively), as was the λ of 0.963 for log number of turns (*P* = 6.55 × 10^-29^ and 3.71 × 10^-05^, respectively). The λ value of 0.995 for log body mass was significantly different from 0 but not from 1 (*P* = 4.09 × 10^-53^ and 0.30, respectively), thus only this trait showed significant phylogenetic signal consistent with Brownian motion (Additional file 1: Table S7 for full results).

## Discussion

We undertook three-dimensional reconstructions of the cochleae of 56 bat species, and compared relative basilar membrane length and number of turns among bats and non-echolocating mammals. By relating these structures to echolocation abilities and call parameters, we assessed whether the acquisition of high-frequency hearing and echolocation in bats was associated with morphological change of the inner ear.

### Cochlear gross morphology

Across the bat order, cochleae were found to have highly variable morphologies, with evidence of both a phylogenetic signal as well as one relating to echolocation ability. The extent of this morphological variation differed greatly across families, with some (e.g. Rhinolophidae and Pteropodidae) displaying low inter-specific differences, and others (e.g. Hipposideridae and Mormoopidae) showing higher variation. This pattern might in part reflect variation in the echolocation call parameters of our focal species (see Figure 2); for example the hipposiderid *Cloeotis percivali* has one of the highest known echolocation call frequencies at ~212 kHz [46], compared to values of 64–157 kHz for other members of this family. The *Cloeotis* cochlea contains a highly modified basal turn compared to the other species studied, and these modifications are consistent with the tonotopic organisation of the cochlea in which the basal area corresponds to the highest frequencies. This gradation is thought to be at least partly achieved by a decrease in stiffness of the basilar membrane from base to apex [14]. Similar to *Cloeotis*, *Pteronotus parnellii* – which along with the Old World horseshoe bats has independently evolved CF echolocation [47] – also shows considerable expansion of the cochlear basal turn (see Figure 2), which probably relates to the well-developed auditory fovea in this taxon (as reviewed by [48]).

### Relative basilar membrane length, number of cochlear turns and echolocation

Basilar membrane lengths also varied widely across the bat species studied. All Old World fruit bats were found to have relative basilar membrane lengths similar to those of non-echolocating mammals. Although echolocating bats typically had elongated basilar membranes compared to non-echolocating mammals, we found no consistent significant difference between these groups after accounting for phylogenetic relatedness. Some echolocating bats showed surprisingly short basilar membranes; for example, *Macroderma gigas* had a similarly proportioned basilar membrane to a marsupial, with values for both species falling below the placental mammal distribution. This is noteworthy given the documented development and elongation of therian inner ear features [5,7], and suggests that more in-depth sampling is necessary to quantify inter-specific variation accurately within groups. Previous studies have also found that some members of the Megadermatidae have small cochleae for their body mass [31], and suggest not all echolocating bats have enlarged cochleae. The number of cochlear turns was found to be typically, but not absolutely, higher in the echolocating bats examined (2 - 3.75 turns) compared to the Old World fruit bats (1.75 – 2.25), and published values for other placental mammals (1.5 – 4.25) (Additional file 1: Table S2; [49,50]).

Basilar membrane length displayed an overall negative allometric relationship with body mass, indicating that small mammals have proportionally longer basilar membranes than large mammals. Previously no relationship was found between absolute basilar membrane length and number of turns [18], however, we found a positive significant correlation after accounting for body mass and phylogeny using Bayesian phylogenetic mixed models. This result suggests that cochlear coiling in order to accommodate the basilar membrane may be more important in small-bodied species. For example, in Old World horseshoe bats, low body mass and long basilar membranes might explain their characteristically high number of cochlear turns.

We found that the number of cochlear turns and basilar membrane length were both correlated with echolocation call parameters after taking phylogeny into account. All measures of echolocation call frequency displayed a significant negative relationship with basilar membrane length, and significant positive association with the number of cochlear turns. Body mass was not found to significantly improve model fit which was somewhat surprising given the previously established relationship between body mass and echolocation call frequency [51,52]. It is as yet unclear whether the inferred hearing frequency is influenced by the number of turns *per se –* perhaps due to some mechanical properties – or whether this result is simply an artefact of the relationship between turn number and membrane length. Previously it was shown that cochlear coiling may be an important factor in determining the lower frequency limit of hearing [20], therefore, potential mechanical implications of cochlear shape on hearing should not be overlooked. In fact, cochlear width, basilar membrane length and number of turns are all likely to be interrelated [8]. The tonotopic organisation of the cochlea means it is not necessarily essential for an animal with good high frequency hearing to have either many turns and/or long basilar membranes; for example, echolocating cetaceans cochleae are not characterised by high numbers of turns but instead have a greatly expanded basal turn [53]. Therefore, given that the optimal cochlear form is likely to vary between different mammalian groups [13], as well as across species comparative studies, within-group comparisons are also critical for understanding inner ear adaptations.

Of the bats we examined, the horseshoe bats (Rhinolophidae) were found to have the longest relative basilar membrane lengths, which is probably an adaptation to their fine auditory tuning to their CF calls. While call frequencies in horseshoe bats have previously been shown to correlate with cochlear width and body mass e.g. [51,52], we found that echolocation call frequencies of three size morphs of *Rhinolophus philippinensis* were lower than expected given their forearm, body mass and basilar membrane length, based on the relationship calculated from other congeners. This supports genetic data suggesting these bats have undergone very recent divergence via call frequency shifts [54], which could lead to a decoupling between call parameters and morphology also see [55].

Our study sought to build on previous methods that attempted to relate inner ear measurements with auditory thresholds in vertebrates e.g. [18,20,56]. These studies have previously focused on taxa considered to have ‘generalist’ hearing, therefore, it is of particular interest to see how these previously documented relationships may apply to species considered to have ‘specialist’ hearing. However, as discussed above, from correlation studies alone it is impossible to make functional inferences, for example, regarding cochlear coiling. Furthermore although a precedent has been set to correlate low frequency hearing with the product of membrane length and number of turns, the functional rationale behind this has not been explained [18,20]. It is therefore vital that as three dimensional inner ear datasets continue to be collected, increasingly sophisticated models are developed that will more accurately reflect the functional aspects of vertebrate auditory systems.

### Ancestral reconstructions and origins of echolocation

Inferred hearing characteristics based on ancestral reconstructions of cochlear morphology suggested that the ancestor of modern bats was likely capable of perceiving higher frequencies than many other mammals [4]. However, the reconstructed upper hearing limit cannot be taken as proof that this species could echolocate, nor even hear well at very high frequencies; in fact, the audiograms of many non-echolocating mammals, including Old World fruit bats, show hearing limits of >40 KHz [57], whereas some species of laryngeal echolocating bats (e.g. some members of Molossidae) utilise low frequency echolocation calls, with either the entire FM sweep or just part of the call, within the frequency range audible to humans [58]. Previous morphological examination of the earliest known fossil bat, *Onychonycteris finneyi*, suggested an inability to echolocate [40]; nevertheless, this taxon was almost certainly not the last common ancestor of the bat ‘crown’ group considered in our study and thus direct comparisons with our results may not be meaningful. Others have attempted to predict the echolocation capabilities of ‘primitive’ fossil bats from their inner ear dimensions [31,59], however, until the exact placement of the early fossil bat taxa with respect to modern bats is resolved [40], then their ability to inform our knowledge of the origin of echolocation remains limited.

A persistent problem with attempts at exploring the correlations between bat inner ears and their auditory capabilities is the lack of published audiograms. As echolocation call frequencies are expected to correlate with hearing capabilities (as previously demonstrated [60]) it should be possible to obtain functionally meaningful correlations between inner ear morphology and echolocation parameters. Yet such associations will be less straightforward in bats that use frequency modulated (FM) echolocation, where single calls might cover a range of frequencies and so make parameterisation difficult. Furthermore, the harmonic structure of echolocation calls must be taken into account, for example, the Old World CF bats, utilise calls with the most energy in the second harmonic [61].

### Rate shifts in inner ear morphology

The only significant rate shift (a decrease) in the rate of morphological evolution was found in the relative membrane length of the ancestral Yangochiroptera. Typically, members of the Yangochiroptera were also shown to have longer relative basilar membrane lengths compared to non-echolocating Old World fruit bats. Therefore, this apparent slowing down could suggest that the increase in basilar membrane length was an adaptive trait from an early point in Yangochiroptera evolution. Given the critical role of the basilar membrane in supporting the organ of Corti (which contains the inner and outer hair cells) it might be expected that these structures will show adaptations for processing ultrasonic echoes. Indeed, three genes (*Tmc1, Kcnq4* and *Pcdh15,*) involved in hair cell structure and function show evidence of positive and/or divergent selection acting on the ancestral Yangochiroptera branch [43-45]. The basilar membranes themselves are also known to be long in echolocating members of the Yinpterochiroptera, although this clade did not display evidence of a consistent decrease in the rate of morphological evolution. Instead we found some evidence of an increase in basilar membrane evolution rate that may be related to recent speciation events in horseshoe bats, especially since this group contains among the youngest taxa in our study. Surprisingly, given their highly modified cochleae, no significant rate shifts were found across all branches of the Old World CF bats, which could reflect poor taxonomic sampling, or might mean that neither of the two morphological characters examined in this study fully describes their modified inner ears. For example, in CF bats the presence of auditory foveae, and more generally basilar membrane width, will both be important considerations for modelling bat cochlear mechanics [29,34,56].

Neither relative basilar membrane length nor the number of turns showed evidence of a positive shift in morphological change on the ancestral branch of all modern bats, as might be expected if the acquisition of sophisticated laryngeal echolocation had occurred rapidly at this stage of bat evolution. Instead echolocation must have evolved either gradually in this branch without showing a detectable elevated rate of morphological change compared to the background rate, or otherwise it could have evolved later in bat evolution following the divergence of the two main suborders (~64 Ma [39,62]). The absence of any rate change in the branch of the non-echolocating Old World fruit bats, as might be expected if there had been a loss, would appear to support the latter scenario also see [39,42-45]. The finding that *O. finneyi* did not possess an enlarged cochlea [40] whereas other Eocene fossil bats did, provides additional evidence that echolocation was not present in all early bat lineages, and might have arisen over a short evolutionary timeframe.

Of the three morphological characters examined (basilar membrane length, number of turns and body mass) for which phylogenetic signal was estimated, only body mass showed consistent variation across the tree (i.e. following Brownian motion, BM). In comparison, observed species values of both inner ear characters deviated from the pattern expected given the phylogeny (branch lengths and topology). Consequently variation shown across the species is not consistent with BM, indicating that certain types of traditional phylogenetic corrections, such as independent contrasts, may not be suitable using the untransformed tree. Given the sophisticated high-frequency hearing possessed by some laryngeal echolocating bats it is perhaps not surprising that the morphological variation of the two inner ear features studied here were found not to fit the pattern expected under BM. Previous studies that have documented morphological variation in the inner ears of key taxa have focused principally on primates, rodents and cetaceans [22,49,63]. In contrast to the results found by this study, it has previously been concluded that primate inner ears have evolved under BM and have been used as informative phylogenetic characters [63,64], so corroborating assertions that primates have unspecialised ‘generalist’ hearing [65].

## Conclusions

Our study focused on two key parameters of the inner ear, both of which have putatively interacted to play a crucial role in the development of the high-frequency sensitivity that is characteristic of mammalian hearing. While the inner ears of Old World non-echolocating fruit bats did not deviate significantly from other non-echolocating mammals, laryngeal echolocating bats were shown to display highly variable cochleae, and correlations with echolocation call parameters suggest that inner ear morphology is mechanistically linked to call structure in echolocating species. At the same time, patterns in trait variation associated with echolocation were not universal across all echolocating species, and, furthermore, some were not robust after taking phylogeny into account. Ancestral reconstructions suggest that the common ancestor of extant bats had well-developed high-frequency hearing; however, shifts in the rate of morphological evolution suggest that significant changes in inner ear morphology occurred after the two main bat suborders diverged, consistent with multiple origins of echolocation in bats. Finally, our study examined morphological variation of the inner ears from ~5% of extant bat diversity based on recent species estimates [66]. Further fine-scale studies are therefore necessary to fully understand the remarkable morphological diversity of the bat order.

## Materials and methods

We studied 56 bat species (*n* = 68 individuals) from 16 families, with broad taxonomic, geographic, ecological and echolocation call type coverage (Additional file 1: Table S1 for species list). Our dataset included three documented size-morphs of *Rhinolophus philippinensis*, which appear to be incipient species [54]. Specimens were scanned in the frontal plane using the Metris X-Tek HMX ST 225 CT System at the Department of Mineralogy, EMMA Division, NHM, London. Volumes were reconstructed using CT PRO (Metris X-Tek, UK), and following reconstruction volumes were visualized using VG Studio Max 2.0 (Volume Graphics, Heidelberg, Germany). Internal voids of bony labyrinth were digitally dissected to produce digital endocasts, and converted into shells describing the surface geometry with MeshLab v.1.2.2 (MeshLab Visual Computing Lab - ISTI - CNR).

### Cochlear morphology

#### 1) Basilar membrane length

Using the ‘single point’ feature in Landmark v3.6 [67], a series of 86 approximately equidistantly placed landmark points were placed along the length of the depression between the *scala media* and the *scala tympani*. These points approximated the position of the outer edge of the basilar membrane, beginning at the lowest point of the base of the basilar membrane (where the depression between the two *scala* is first visible), and ending at the apex of the cochlea (Additional file 5: Figure S1A). This number of landmarks should adequately describe the path of the membrane (Additional file 5: Figure S1B). The 3D coordinates were exported into Microsoft Excel, where the total Euclidean distance was calculated by summing the distance between each set of consecutive points. Where the distance (x) between points (*p*_1_, *p*_2_, *p*_3_) and (*q*_1_, *q*_2_, *q*_3_) is calculated using the formula:

$$x=\sqrt{{\left(p1-q1\right)}^{2}+{\left(p2-q2\right)}^{2}+{\left(p3-q3\right)}^{2}}$$

The measurements collected by this study were combined with those from previous studies (Additional file 1: Table S2 for values and sources). In order to compare the relationship between the linear measurements of basilar membrane length and body mass, the cube root of the latter was calculated. Values were then log_10_ transformed so that the linear regression between the variables could be studied. To test whether data from bats fell into the same distribution as published data from non-bats, we calculated the 95% prediction intervals (PI) of the latter.

#### 2. Number of cochlear spiral turns

The number of cochlear turns was measured in each bat species following West [18], in which the cochlea was viewed apically and a line drawn from the point of the round window (where the cochlear duct initially begins to curl) to the apex. The number of times the line was crossed by the path of the duct was then recorded. Measurements were taken to the nearest one quarter of a complete turn. We supplemented our measurements with those from literature sources to include additional bat and non-bat species (Additional file 1: Table S2).

#### Constructing the phylogeny and estimating branch lengths

Published c*ytochrome b* sequences, for 131 ingroup species and 3 outgroup species (Additional file 1: Table S2) were aligned using ClustalW2 [68] and checked by eye. Branch lengths were estimated using a constrained tree topology based on published phylogenies [69-82]. *Cytochrome b* sequences were not available for most members of Megadermatidae; therefore we used the phylogeny proposed by Griffiths et al*.*[83]. We were able to estimate diversification times for the division between *Macroderma gigas* with *Megaderma lyra,* and this value and those from Jones et al. [84] were used to date the remaining nodes. Nine fossil calibration points were used (Additional file 1: Table S3), each following a normal prior distribution with mean and standard deviation set so that the 5^th^ and 95^th^ quantiles correspond to the published lower and upper suggested node ages respectively. Analyses were run in BEAST v.1.5.4 using an uncorrelated log-normal relaxed molecular clock [85], a Yule speciation prior and a GTR+I+Γ model, for 10,000,000 generations, with every 1000 parameters logged. Tracer v.1.5 was used to check for appropriate burn-in length and run convergence. The maximum clade credibility tree was produced using TreeAnnotator v.1.5.4, with a sample burn-in of 500 and node heights set to mean-heights.

To test whether observed inter-species variation in cochlear parameters remained after accounting for phylogenetic relatedness, we implemented Bayesian phylogenetic mixed models (BPMMs) in ‘MCMCglmm’ [86] in R v.2.11.1 ( http://www.R-project.org) (see Additional file 1: Supplementary Methods). Model fit was assessed based the Deviance Information Criterion (DIC) in which ΔDIC values of ≥2 was used to denote significant statistical improvement. For significance tests of fixed effects, we report the *P*_MCMC_ value, which is twice the posterior probability that a model parameter is greater or less than zero (whichever is lower), as estimated by the Markov chain, and is one possible Bayesian analogue to a two-tailed frequentist p-value.

### Inferring ancestral auditory and echolocation capabilities

Ancestral reconstructions of relative basilar membrane length and number of cochlear turns were estimated using the maximum likelihood approach, ‘ace’ function, and phylogenetic independent contrasts, both undertaken in the R package ‘PICANTE’ [87]. Low and high frequency auditory thresholds, at 30 and 60 decibels (dB), were extracted from published audiograms for 14 bat and 24 non-bat mammal species (Additional file 1: Table S4). The estimated body mass of the ancestral bat, 19.22 g, was taken from Safi et al. [88]. The upper frequency hearing limits of the hypothetical ancestral bat at 30 and 60 dB were estimated using the relationship between log relative membrane length and hearing limits in extant taxa. Lower frequency hearing limits at 30 and 60 dB were estimated using the relationship between the product of log basilar membrane length and the number of cochlear turns with low frequency hearing limits in extant taxa, following published methods [18,20].

Additionally, to test for an association between bat inner ear morphology and echolocation call frequency, we collected values for three echolocation call parameters: mean frequency at maximum intensity, minimum frequency and maximum frequency. Due to species specific differences in call intensities e.g. [89,90], echolocation calls frequencies are not recorded at consistent sound levels, and instead by convention the absolute minimum, maximum and peak energy frequencies are typically measured. The audiograms that are available for echolocating bat species suggest that the bats are capable of hearing the majority, if not the entire, range of frequencies covered by the call e.g. [91]. As many values as possible were collected for each echolocation call parameter, and in cases where more than one value was available, we took the mean.

### Rates of morphological change

To assess the relative support for the two scenarios proposed to explain the absence of echolocating in Old World fruit bats, we characterised the rate of cochlear evolution (based on coils and length) across the Laurasiatheria. For this we performed a Bayesian analysis of rate shifts in the morphological traits using the package ‘AUTEUR’ [92]. Analyses were run twice, with 4,000,000 generations sampled every 4,000 generations. Convergence was assessed using Tracer v1.5 [93]. Shift plots were drawn with a burn in of 25%. In this analysis, each branch of the phylogeny is coloured according to the calculated model-averaged rate estimate for that particular branch and the posterior probability of a rate shift occurring at each node is also indicated. For our study we defined significant rate shifts as those with a posterior probability greater than 0.95.

As a further test of character evolution, the phylogenetic signal (Pagel’s λ) of basilar membrane length, body mass and the number of cochlear turns was estimated using the ‘fitContinuous’ argument of the ‘Geiger’ package [94]. The estimated values were then tested to see if they were significantly different to either 0 or 1, where a λ estimate of 1 signifies an exact fit between the phylogeny and a given characters under Brownian motion, and a λ of 0 signifies no phylogenetic signal and thus all species approximate independent points.

### Availability of supporting data

The data sets supporting the results of this article are included within the article and its additional files.

## Competing interests

The authors declare that they have no competing interests.

## Authors’ contributions

KTJD and SJR conceived and designed the study. KTJD undertook data collection and analysed the data with input from SJR. IM provided material. KTJD and SJR wrote the paper with input from IM. All authors read and approved the final manuscript.

## Supplementary Material

Additional file 1 Supplementary material. Click here for file

Additional file 2: Figure S2 Multiple regression plots of echolocation call parameters, basilar membrane length, number of cochlear turns and body mass. Stepwise multiple regressions suggest that equations with only inner ear parameters were the best fitting models: log maximum frequency = -0.96 log basilar membrane + 1.58 log turns + 2.22, multiple *R*^*2*^= 0.26, F = 9.01 (2, 51 d.f.), *P* = 4 x 10^-4^; log peak energy frequency = -1.01 log basilar membrane + 2.26 log turns + 1.92, multiple *R*^*2*^= 0.36, F = 14.05 (2, 51 d.f.), *P* = 1.38 x 10^-5^; log minimum frequency = -1.02 log basilar membrane + 2.76 log turns + 1.60, multiple *R*^*2*^= 0.40, F = 17.09 (2, 51 d.f.), *P* = 2.09 x 10^-6^. Click here for file

Additional file 3: Figure S3 Maximum likelihood ancestral reconstructions of bat inner ears - (**A**) relative basilar membrane length and (**B**) number of cochlear turns. Phylogenies and character values are depicted as ‘Traitgrams’, whereby the position along the y-axis corresponds to node age in millions of years and position along the x-axis corresponds to the reconstructed character value. Coloured bars indicate key subdivisions within bats: Old World fruit bats (orange); echolocating Yinpterochiroptera (blue); Yangochiroptera (green). Keys nodes: bat common ancestor (**a**); Yangochiroptera common ancestor (**b**); Yinpterochiroptera common ancestor (**c**); Old World fruit bat common ancestor (**d**); echolocating Yinpterochiroptera common ancestor (**e**). Click here for file

Additional file 4: Figure S4 Morphological parameters versus echolocation call frequency in *Rhinolophus* species. Published values for species taken from literature (black points), the three *Rhinolophus philippinensis* size morphs measured by this study: small (red), medium (orange), large (yellow) and values published for one *R. philippinensis* values taken from [28] (blue). (**A**) Average forearm length, body mass and echolocation call frequency for *Rhinolophus spp*. from values obtained from literature sources. A significant negative relationship was found (log CF = -1.54 log forearm + 4.44; *R*^*2*^ = 0.42, F = 36.57, *P* = 1.98 x 10^-7^ and log CF = -0.38 log body mass + 2.24; *R*^*2*^ = 0.25, F = 12.34, *P* = 0.001). (**B**) The relationship between basilar membrane length and constant frequency echolocation call (log CF = -0.98 log basilar membrane + 3.09; *R*^*2*^ = 0.63, F *=* 21.74, *P <* 0.001). Click here for file

Additional file 5: Figure S1 Measuring basilar membrane length from reconstructed inner ear endocasts. (**A**) Left: Medial view of the right cochlear endocast of *Craseonycteris thonglongyai* (specimen number HZM.1.34982, ref. Table S1). A representation of the path of the basilar membrane measured by this study is shown by the dotted line. Right upper: Apical view of the right cochlear endocast of *C. thonglongyai* (HZM.1.34982). Black arrows correspond to the end point of the representation of the path of the basilar membrane (dotted line) measured by this study. Right lower: Medial view of the right cochlear endocast of *C. thonglongyai* (HZM.1.34982). Black arrows correspond to the start point of the representation of the path of the basilar membrane (dotted line) measured by this study. (**B**) Two-dimensional plots showing the representative paths of the basilar membrane for the right cochlea of *Pipistrellus pipistrellus,* using either 86 or 43 landmark points, connected with straight connecting lines. The basilar membrane path as depicted by a smoothed curvilinear path is also superimposed over these points. The estimated length calculated from the subset of 43 coordinates was only 8.304 mm, compared to 8.473 mm from 86 coordinates. This corresponds to a negative difference of 0.170 mm or a 2% underestimate of membrane length. Furthermore, the path traced by the straight lines connecting the 86 points much more faithfully follows that of the curved path. Therefore, 86 landmark points were deemed to be a suitable compromise between efficiency and accuracy and was used to collect all basilar membrane estimates. The 86 landmarks used in this study (circles); curved path between points (black line); straight lines between points used to estimate basilar membrane length (dotted line); 43 points (white circles), and the dashed line the straight line distance between white circles (dashed line). Click here for file
