# Supplementary material for: Evolutionary origins of ultrasonic hearing and laryngeal echolocation in bats inferred from morphological analyses of the inner ear
Source: Front Zool. 2013 Jan 30;10:2. doi: 10.1186/1742-9994-10-2 (PMC3598973; doi:10.1186/1742-9994-10-2)
Supplement: Additional file 1 — Supplementary material. [file 1742-9994-10-2-S1.doc]

**SUPPLEMENTARY MATERIAL**

**Evolutionary origins of ultrasonic hearing and laryngeal echolocation in bats inferred from morphological analyses of the inner ear**

Kalina T.J. Davies, Ibnu Maryanto, Stephen J. Rossiter

**Supplementary Information**

**Materials and Methods**

*Study sample and acquisition of µCT-scan data*

In total 68 specimens, representing 56 bat species from 16 families, were obtained for this study (see Table S1). Species were prioritised based on maximising taxonomic and geographic coverage, ecological diversity, behavioural differences and echolocation call type. Preservation state and method of preparation varied between specimens; seven were complete specimens preserved in ethanol, the remaining specimens being prepared skulls. Adult specimens were used, apart from *Hypsignathus monstrosus* where the only available specimen was a female sub-adult. We were unable to control for specimen gender, and, therefore, as with previous studied we assumed that sexual dimorphism and individual asymmetry in mammalian inner ears were negligible [1, 2]. In order to make functional interpretations based on representative individuals it was assumed that inter-specific variation is greater then intra-specific variation.

Each specimen was scanned in the frontal plane using the Metris X-Tek HMX ST 225 Computer Tomography (CT) System at the Department of Mineralogy, EMMA Division, The Natural History Museum, London. The complete basal part of the skull was scanned so as to include both inner ears, with the exception of specimen NHM.65.3990, where the single remaining bony labyrinth was dislodged from the skull and was thus scanned directly. Following calculation of the centre of rotation and cropping the region to reconstruct to the desired region of interest, volumes were constructed using CT PRO (Metris X-Tek, UK). Reconstructed volumes were then visualized using VG Studio Max 2.0 (Volume Graphics, Heidelberg, Germany). The voxel size of each specimen is listed in Table S1.

***Old and New World constant-frequency bats***

For the Old World constant frequency (CF) bats (Rhinolophidae and Hipposideridae) and the New World *Pteronotus parnellii* (Mormoopidae) a relationship between basilar membrane length, body size and call frequency was predicted. This was investigated by examining the published frequency of maximum energy (ie. the CF component of the call) and morphological parameters for 53 species from the Rhinolophidae, six from the Hipposideridae, and *P. parnellii* (see Tables S2, S8 and S9). Specimens examined from the Rhinolophidae included three documented size morphs of *R. philippinensis* (Small morph: location = Buton, male, 47.4 mm (forearm length), 6.5 g (mass); Large morph, location = Buton, male, 55.3 mm, 12.0 g; Intermediate morph, location = Kabaena, female, 48.4 mm, 7.0 g). These morphs have been reported to have undergone recent speciation via call frequency shifts along a harmonic series [3] and, therefore, might be expected to deviate from any overall relationship between physical features and call-frequency in this family. All echolocation and morphological variables were logged transformed to equalise variances and normalise data.

***Accounting for phylogenetic relatedness***

To control for the shared ancestry of characters, and thus determine whether differences in inner ear morphology were maintained after accounting for phylogenetic relatedness, we used Bayesian phylogenetic mixed models (BPMMs) implemented in ‘MCMCglmm’ [4] in R v.2.11.1. This analysis accounts for phylogenetic relatedness in the context of a linear mixed effects model – at type of linear statistical model that contains both fixed and random effects. Here, the random effect is the covariance between taxa caused by their shared evolutionary histories, as implied by the phylogeny for the taxa. Within these models, the contribution of ‘shared evolutionary history’ is included in the model as an explanatory variable for the trait of interest, so that the effect of other traits can be analysed correctly in the presence of this non-independence. The structure of these models is identical to that of the ‘animal model’ widely used in animal breeding and quantitative genetics, a fact that can be exploited to make use of software and statistical approached developed to fit these complex models efficiently and flexibly, for example with non-Gaussian response variables [5].

All analyses were run with the same basic settings: animal as a random effect with pedigree information obtained from the dated phylogeny, and with proper priors with a low degree of belief (n = 1) and phenotypic trait variance divided equally between genetic and residual effects. All models were run with 1,000,000 iterations with 100,000 burn in and thinning interval of 100. Autocorrelation values <0.01 were used to check for good mixing. To compare models utilising phylogenetic information, we examined the Deviance Information Criterion (DIC); with DIC values ≥2 taken to indicate significantly improved model fit. For tests of fixed effects, we report the *P*_MCMC_ value, which is twice the posterior probability that a model parameter is greater or less than zero (whichever is lower), as estimated by the Markov chain, and is one possible Bayesian analogue to a two-tailed frequentist p-value.

***Constructing the phylogeny and estimating branch lengths***

*Cytochrome* *b* sequences were obtained for 131 ingroup and 3 outgroup species (see Table S2 for species names, GenBank accession numbers and sources). For seven species, sequences from a congeneric substitute were used if those from the actual species were not available. In the cases of multiple haplotypes, a representative sequence was chosen at random. Nucleotide sequences were aligned using ClustalW2 [6] and checked by eye. The alignment was imported into BEAUti v. 1.5.4 [7], this utility was used to produce the correctly formatted input file (xml-file) for BEAST v.1.5.4. A user-specified starting tree was used, based on the published phylogenies [8-21]. This topology was enforced throughout the analysis by removal of four operators which effect topological rearrangements. Nine fossil calibration points were used (see Table S3), which each following a normal prior distribution with mean and standard deviation set so that the 5^th^ and 95^th^ quantiles correspond to the published lower and upper suggested node ages respectively. Analyses were run in BEAST v.1.5.4 using an uncorrelated log-normal relaxed molecular clock [22], a Yule speciation prior and a GTR+I+Γ model, for 10,000,000 generations, with every 1000 parameters logged. Tracer v.1.5 was used to check for appropriate burn-in length and run convergence. The maximum clade credibility tree was produced using TreeAnnotator v.1.5.4, with a sample burn-in of 500 and node heights set to mean-heights. Currently the phylogenetic relationship of the Megadermatidae remains uncertain; so far two conflicting alternative topologies have been suggested based only on morphological characters [23, 24]. For this group, we attempted to construct a molecular phylogeny using NADH dehydrogenase subunit 2 (ND2) gene sequences from GenBank for all available Megadermatidae [*Macroderma gigas* (AY504541.1); *Cardioderma cor* (AY504540.1); *Megaderma lyra* (AY504542.1) and *Megaderma spasma* (AY504543.1)] and three out-group taxa [*Rhinopoma hardwickei* (AY504545.1); *Hipposideros cineraceus* (AY504529.1); *Hipposideros bicolor* (AY504527.1)]. The TrN+I+G model of substitution, selected using jModelTest v0.1.1 [25], was implemented in MEGA v5 [26] to construct a maximum likelihood phylogeny with 1000 bootstraps. However, nodes within Megadermatidae were poorly supported (bootstrap support ≤ 50) and therefore, could not be resolved (results not shown). Therefore, this study follows the topology of Griffiths et al. [24], with diversification times based on our results from *Cytochrome b* for the division between *M. gigas* with *M. lyra,*  and Jones et al*.* [27].

**Supplementary results**

A significant negative relationship was found between call frequency and two measures of body size, forearm length and body mass, for values from 52 *Rhinolophus* *spp*. obtained from literature sources (log CF = -1.54 log forearm + 4.44; R^2^ = 0.42, F = 36.57, *P* = 1.98 x 10^-7^ and log CF = -0.38 log body mass + 2.24; R^2^ = 0.25, F = 12.34, *P* = 0.001). Echolocation call frequencies for *R.* *philippinensis* small and medium morphs fell within the 95% PI for forearm length (see Figure S4a). Furthermore a significant relationship was found between basilar membrane length and echolocation call frequency log CF = -0.98 log basilar membrane + 3.09; R^2^ = 0.634, F *=* 21.74, *P <* 0.001). The call frequencies of the medium and large morph fell within the 95% PI for basilar membrane length, though the small morph was on this line (see Figure S4b). Both regressions suggest lower frequencies in the *R.* *philippinensis* morphs than expected for either their respective forearm or basilar membrane length.

**Supplementary Figure legends**

**Supplementary Figure S1**

**Measuring basilar membrane length from reconstructed inner ear endocasts.**

(A) Left: Medial view of the right cochlear endocast of *Craseonycteris thonglongyai* (specimen number HZM.1.34982, ref. Table S1). A representation of the path of the basilar membrane measured by this study is shown by the dotted line.

Right upper: Apical view of the right cochlear endocast of *C. thonglongyai* (HZM.1.34982). Black arrows correspond to the end point of the representation of the path of the basilar membrane (dotted line) measured by this study.

Right lower: Medial view of the right cochlear endocast of *C. thonglongyai* (HZM.1.34982). Black arrows correspond to the start point of the representation of the path of the basilar membrane (dotted line) measured by this study.

(B) Two-dimensional plots showing the representative paths of the basilar membrane for the right cochlea of *Pipistrellus pipistrellus,* using either 86 or 43 landmark points, connected with straight connecting lines. The basilar membrane path as depicted by a smoothed curvilinear path is also superimposed over these points. The estimated length calculated from the subset of 43 coordinates was only 8.304 mm, compared to 8.473 mm from 86 coordinates. This corresponds to a negative difference of 0.170 mm or a 2 % underestimate of membrane length. Furthermore, the path traced by the straight lines connecting the 86 points much more faithfully follows that of the curved path. Therefore, 86 landmark points were deemed to be a suitable compromise between efficiency and accuracy and was used to collect all basilar membrane estimates. The 86 landmarks used in this study (circles); curved path between points (black line); straight lines between points used to estimate basilar membrane length (dotted line); 43 points (white circles), and the dashed line the straight line distance between white circles (dashed line).

**Supplementary Figure S2**

**Multiple regression plots of echolocation call parameters, basilar membrane length, number of cochlear turns and body mass.** Stepwise multiple regressions suggest that equations with only inner ear parameters were the best fitting models: log maximum frequency = -0.96 log basilar membrane + 1.58 log turns + 2.22, multiple *R^2^*= 0.26, F = 9.01 (2, 51 d.f.), *P* = 4 x 10^-4^; log peak energy frequency = -1.01 log basilar membrane + 2.26 log turns + 1.92, multiple *R^2^*= 0.36, F = 14.05 (2, 51 d.f.), *P* = 1.38 x 10^-5^; log minimum frequency = -1.02 log basilar membrane + 2.76 log turns + 1.60, multiple *R^2^*= 0.40, F = 17.09 (2, 51 d.f.), *P* = 2.09 x 10^-6^.

**Supplementary Figure S3**

**Maximum likelihood ancestral reconstructions of bat inner ears -** (A) relative basilar membrane length and (B) number of cochlear turns. Phylogenies and character values are depicted as ‘Traitgrams’, whereby the position along the y-axis corresponds to node age in millions of years and position along the x-axis corresponds to the reconstructed character value. Coloured bars indicate key subdivisions within bats: Old World fruit bats (orange); echolocating Yinpterochiroptera (blue); Yangochiroptera (green). Keys nodes: bat common ancestor (a); Yangochiroptera common ancestor (b); Yinpterochiroptera common ancestor (c); Old World fruit bat common ancestor (d); echolocating Yinpterochiroptera common ancestor (e).

**Supplementary Figure S4**

**Morphological parameters versus echolocation call frequency in *Rhinolophus* species.** Published values for species taken from literature (black points), the three *Rhinolophus philippinensis* size morphs measured by this study: small (red), medium (orange), large (yellow) and values published for one *R.* *philippinensis* values taken from [28] (blue).

(A) Average forearm length, body mass and echolocation call frequency for *Rhinolophus* *spp*. from values obtained from literature sources. A significant negative relationship was found (log CF = -1.54 log forearm + 4.44; *R^2^* = 0.42, F = 36.57, *P* = 1.98 x 10^-7^ and log CF = -0.38 log body mass + 2.24; *R^2^* = 0.25, F = 12.34, *P* = 0.001).

(B) The relationship between basilar membrane length and constant frequency echolocation call (log CF = -0.98 log basilar membrane + 3.09; *R^2^* = 0.63, F *=* 21.74, *P <* 0.001).

**Supplementary tables**

**Table S1 Specimen and scan details of the bat species used in this study.**

| Family | Species | Voxel size | Specimen code |
| --- | --- | --- | --- |
| Pteropodidae | *Pteropus rodricensis* | 0.017539 | NHM. 76.3.15.14^1^ |
|  | *Pteropus sp.* | 0.0167 | N/A |
|  | *Rousettus lanosus* | 0.0118 | HZM.16.36082^2^ |
|  | *Rousettus aegyptiacus* | 0.0101 | HZM.107.11626^2^ |
|  | *Hypsignathus monstrosus* | 0.0126 | HZM.1.3518^2^ |
| Rhinolophidae | *Rhinolophus philippinensis (Small)* | 0.0064 | MZB22913^3^ |
|  | *Rhinolophus philippinensis (Medium)* | 0.0073 | MZB22897^3^ |
|  | *Rhinolophus philippinensis (Large)* | 0.0101 | MZB22910^3^ |
|  | *Rhinolophus ferrumequinum* | 0.0073 | HZM.58.20697^2^ |
|  | *Rhinolophus megaphyllus* | 0.0087 | NHM.1903.8.3.3^1^ |
|  | *Rhinolophus pearsonii* | 0.0081 | N/A^2^ |
|  | *Rhinolophus pearsonii* | 0.0083 | HZM.14.35.110^2^ |
|  | *Rhinolophus marshalli* | 0.0106 | N/A^2^ |
|  | *Rhinolophus affinis* | 0.0088 | HZM.29.35223^2^ |
| Hipposideridae | *Hipposideros ridleyi* | 0.0076 | NHM.1983.423^1^ |
|  | *Hipposideros fulvus* | 0.007 | HZM.3.28778^2^ |
|  | *Hipposideros gigas* | 0.0108 | HZM.3.1164^2^ |
|  | *Cloeotis percivali* | 0.0069 | HZM.13.4765^2^ |
|  | *Cloeotis percivali* | 0.0049 | NHM.66.5456^1^ |
| Rhinopomatidae | *Rhinopoma microphyllum* | 0.0082 | NHM.1968.453^1^ |
|  | *Rhinopoma hardwickii* | 0.0073 | HZM.37.9152^2^ |
| Megadermatidae | *Macroderma gigas* | 0.0083 | NHM.1892.5.20.2^1^ |
|  | *Cardioderma cor* | 0.0124 | NHM.1975.2453^1^ |
|  | *Megaderma spasma* | 0.0069 | NHM.1912.11.28.32^1^ |
|  | *Lavia frons* | 0.0085 | HZM.30.25025^2^ |
| Craseonycteridae | *Craseonycteris thonglongyai* | 0.0057 | HZM.1.34982^2^ |
| Miniopteridae | *Miniopterus schreibersii* | 0.0049 | NHM.62.1443^1^ |
|  | *Miniopterus schreibersii* | 0.0085 | HZM.247.22505^2^ |
| Vespertilionidae | *Murina tubinaris* | 0.0106 | N/A^2^ |
|  | *Myotis muricola* | 0.0097 | N/A^2^ |
|  | *Murina suilla* | 0.0069 | NHM.18.79.11.15.16^1^ |
|  | *Murina cyclotis* | 0.0118 | NHM.16.3.25.29^1^ |
|  | *Myotis lucifugus* | 0.005188 | NHM.7.7.7.3359^1^ |
|  | *Scotophilus kuhlii* | 0.0118 | N/A^2^ |
|  | *Scotomanes ornatus* | 0.0097 | N/A^2^ |
|  | *Lasiurus borealis* | 0.0057 | HZM.4.3341^2^ |
|  | *Plecotus auritus* | 0.0055 | N/A |
|  | *Plecotus auritus* | 0.0055 | N/A |
|  | *Plecotus auritus* | 0.0058 | N/A |
|  | *Pipistrellus pipistrellus* | 0.005 | N/A |
|  | *Pipistrellus pipistrellus* | 0.005 | N/A |
|  | *Pipistrellus pipistrellus* | 0.0049 | N/A |
|  | *Pipistrellus pipistrellus* | 0.0047 | N/A |
| Noctilionidae | *Noctilio leporinus* | 0.0118 | NHM.1928.7.21.35^1^ |
|  | *Noctilio leporinus* | 0.0107 | HZM.12.15977^2^ |
| Thyropteridae | *Thyroptera sp.* | 0.007 | N/A |
| Mormoopidae | *Pteronotus (chilonycteris) macleayi grisea* | 0.004548 | NHM.65.3990^1^ |
|  | *Pteronotus parnellii* | 0.0112 | HZM.5.21236^2^ |
|  | *Pteronotus davyi* | 0.0083 | HZM.8.16031^2^ |
|  | *Mormoops megaphylla* | 0.0074 | HZM.2.16030^2^ |
| Phyllostomidae | *Artibeus jamaicensis* | 0.0118 | NHM.1907.1.1.684^1^ |
|  | *Tonatia silvicola* | 0.0101 | NHM.1954.322^1^ |
|  | *Trachops cirrhosus* | 0.0101 | NHM.1924.3.1.33^1^ |
|  | *Desmodus rotundus* | 0.0099 | HZM.41.11631^2^ |
|  | *Anoura geoffroyi* | 0.0063 | NHM.1914.5.21.4^1^ |
|  | *Carollia perspicillata perspicillata* | 0.0079 | HZM.140.29127^2^ |
|  | *Centurio senex* | 0.0088 | HZM.1.13198^2^ |
| Molossidae | *Cheiromeles torquatus* | 0.0186 | NHM.1844.10.17.7^1^ |
|  | *Molossus molossus* | 0.0096 | HZM.61.28841^2^ |
|  | *Tadarida brasiliensis* | 0.006 | NHM.1960.482^1^ |
| Nycteridae | *Nycteris thebaica* | 0.0085 | HZM.214.35929^2^ |
| Natalidae | *Natalus stramineus saturates* | 0.0065 | HZM.8.7055^2^ |
| Emballonuridae | *Taphozous peli* | 0.0186 | HZM.2.18450^2^ |
|  | *Taphozous melanopogon* | 0.0086 | HZM.18.30235^2^ |
|  | *Peropteryx macrotis* | 0.0059 | HZM.3.18512^2^ |
|  | *Saccopteryx bilineata* | 0.0087 | HZM.8.16002^2^ |
|  | *Rhynchonycteris naso* | 0.0057 | HZM.34.5860^2^ |

Origin of specimens:

1 Natural History Museum, London;

2 The Harrison Institute, Sevenoaks;

3 Museum Zoology Bogor, Indonesia;

Abbreviations: N/A – not applicable.

**Table S2**

**Morphological parameters and *Cytochrome b* sequence information for taxa included in this study.**

| Family | Species | Basilar membrane (mm)  Mean ± SD,  Min – Max (n) | Source: | Number of turns | Source: | Body mass (g) | Source: | GenBank  accession number: |
| --- | --- | --- | --- | --- | --- | --- | --- | --- |
| Pteropodidae | *Pteropus rodricensis* | N/A | N/A | 1.75 | This study | 350 | [29] | FJ561392.1 |
|  | *Pteropus sp.* | 16.24 | This study | 1.75 | This study | 1180* | [30] | JN398212.1  (*Pteropus vampyrus*) |
|  | *Pteropus giganteus* | N/A | N/A | 2 | [31] | 1180 | [30] | FJ561381.1 |
|  | *Rousettus lanosus* | 12.52 | This study | 1.75 | This study | 140 | [30] | AF044661.1 |
|  | *Rousettus aegyptiacus* | 11.01 | This study | 1.75 | This study | 140 | [30] | AB085740.1 |
|  | *Hypsignathus monstrosus* | 13.08 | This study | 2.25 | This study | 427 | [30] | DQ445706.1  (*Epomophorus wahlbergi*) |
| Rhinolophidae | *Rhinolophus philippinensis (Small)* | 12.98 | This study | 3.25 | This study | 6.5 | SJ Rossiter, T Kingston | SJ. Rossiter unpub. |
|  | *Rhinolophus philippinensis (Medium)* | 24.67 | This study | 3.25 | This study | 7 | SJ Rossiter, T Kingston | GU724957.1 |
|  | *Rhinolophus philippinensis (Large)* | 29.96 | This study | 3.25 | This study | 12 | SJ Rossiter, T Kingston | SJ. Rossiter unpub. |
|  | *Rhinolophus ferrumequinum* | 17.29 ± 1.69  16.10-18.49 (2) | This study, [32] | 3.25 | This study | 22.6 | [30] | DQ351847.1 |
|  | *Rhinolophus megaphyllus* | 28.64 | This study | 3.75 | This study | 9.8 | [30] | FJ185207.1 |
|  | *Rhinolophus pearsonii* | 21.42 ± 0.01  21.40 – 21.42 (2) | This study | 3.25 - 3.5 | This study | 8.8 | HZM | EF544424.1 |
|  | *Rhinolophus marshalli* | 21.53 | This study | 3.25 | This study | 6.4 | [33] | EU434938.1 |
|  | *Rhinolophus affinis* | 21.99 | This study | 3.5 | This study | 12.6 | HZM | EF544421.1 |
|  | *Rhinolophus rouxi* | 15.6 | [34] | N/A | N/A | 12.57 | [34] | HM134916.1  (*Rhinolophus sinicus*) |
| Hipposideridae | *Hipposideros ridleyi* | 13.86 | This study | 2.75 | This study | 9.3 | [35] | DQ054812.1 |
|  | *Hipposideros fulvus* | 9.61 ± 1.14  8.80 - 10.41 (2) | This study, [36] | 2.75 | This study | 9.25 ± 1.06  8.50 – 10.00 (2) | [37],  [36] | DQ054807.1  (*Hipposideros ater*) |
|  | *Hipposideros gigas* | 22.58 | This study | 3.25 | This study | 89 | [30] | EU934470.1 |
|  | *Cloeotis percivali* | 9.41 | This study | 2.75 | This study | 4.05 | [38] | FJ457616.1 |
|  | *Hipposideros speoris* | 9.55 ± 0.50  9.20 - 9.90 (2) | [36, 39] | N/A | N/A | 11.53 ± 2.17  10.00 - 13.07 (2) | [36, 39] | DQ680823.1 |
|  | *Hipposideros bicolor* | 9.13 | [9] | 2.5 | [9] | 13.29 | [39] | JF320685.1 |
|  | *Hipposideros pomona* | N/A | N/A | 3 | [40] | N/A |  | EU434950.1 |
| Rhinopomatidae | *Rhinopoma microphyllum* | 12.97 | This study | 2.25 | This study | 32 | [30] | DQ337502.1 |
|  | *Rhinopoma hardwickii* | 12.23 ± 0.613  11.8 – 12.67 (2) | This Study, [39] | 2.5 | This study | 12.93 ± 4.77  9.56 – 16.30 (2) | [30, 39] | DQ337494.1 |
| Megadermatidae | *Macroderma gigas* | 6.16 | This study | 2.25 | This study | 123 | [30] | NA |
|  | *Cardioderma cor* | 14.90 | This study | 2 | This study | 28 | [41] | NA |
|  | *Megaderma spasma* | 9.66 | This study | 2.25 | This study | 27 | [35] | AY057942.1 |
|  | *Megaderma lyra* | 9.9 | [36, 39] | N/A | N/A | 41.38 ± 8.94  35.36 – 48.00 (2) | [36, 39] | DQ888678.1 |
|  | *Lavia frons* | 12.60 | This study | 2.25 | This study | 34.8 | HZM | NA |
| Craseonycteridae | *Craseonycteris thonglongyai* | 10.54 | This study | 2.5 | This study | 2.5 | HZM | EF035012.1 |
| Miniopteridae | *Miniopterus schreibersii* | 10.42 ± 0.12  10.33 – 10.50 (2) | This study | 2.25 | This study | 14.20 | [30] | EF530348.1 |
| Vespertilionidae | *Murina tubinaris* | 11.81 | This study | 2.75 | This study | 4.75 | [35] | GQ168904.1 |
|  | *Myotis albescens* | N/A | N/A | 2.5 | [31] | N/A | N/A | AF376839.1 |
|  | *Myotis muricola* | 10.23 | This study | 2.5 | This study | 4.85 | [42] | AY665144.1 |
|  | *Murina suilla* | 10.56 | This study | 2.75 | This study | 3.90 | [35] | GQ168905.1 |
|  | *Murina cyclotis* | 9.64 | This study | 2.5 | This study | 11.30 | [35] | GQ168916.1 |
|  | *Myotis lucifugus* | 8.45 ± 0.93  6.90 – 8.21 (2) | This study, [36] | 2.25 | This study | 7.00 | [36] | AF376854.1 |
|  | *Scotophilus kuhlii* | 13.42 | This study | 2.75 | This study | 23.0 | [43] | EU750931.1 |
|  | *Scotomanes ornatus* | 12.04 | This study | 2.25 | This study | 21.8 | [43] | DQ435069.1 |
|  | *Lasiurus borealis* | 10.83 | This study | 2.5 | This study | 16.7 | [30] | NC_016873.1 |
|  | *Plecotus auritus* | 9.09 ± 0.29  8.76 – 9.30 (3) | This study | 2 | This study | 9.00 | [30] | EF570882.1 |
|  | *Pipistrellus pipistrellus* | 8.89 ± 0.32  8.47 – 9.23 (4) | This study | 2.5 | This study | 6.55 | [44] | AJ504443.1 |
|  | *Nyctalus noctula* | 11.8 | [39] | N/A | N/A | 5.19 | [39] | DQ120873.1 |
|  | *Eptesicus serotinus* | 8.9 | [39] | N/A | N/A | 22.30 | [39] | AF376837.1 |
| Noctilionidae | *Noctilio leporinus* | 16.27 ± 0.35  16.03 – 16.52 (2) | This study | 2.75 | This study | 59 | [30] | AF330801.1 |
| Thyropteridae | *Thyroptera sp.* | 13.42 | This study | 2.5 | This study | 3.5 | [30] | AY621005.1 |
| Mormoopidae | *Pteronotus (chilonycteris) macleayi grisea* | 11.77 | This study | 3 | This study | 5.35 | [45] | AY604461.1 |
|  | *Pteronotus parnellii* | 15.75 ± 1.27  14.30 – 16.69 (3) | This study | 2.5 | This study | 14.57 ± 2.51  12.00 – 17.02 (3) | [39, 46] | AY604456.1 |
|  | *Pteronotus davyi* | 11.42 | This study | 2.75 | This study | 10.90 | [30] | AF338671.1 |
|  | *Mormoops megaphylla* | 13.21 | This study | 2.5 | This study | 17.00 | [30] | AF330808.1 |
|  | *Chilonycteris rubiginosa (Pteronotus parnellii?)* | N/A | N/A | 3 | [47] | N/A | N/A | AY604457.1 |
| Phyllostomidae | *Artibeus jamaicensis* | 12.96 | This study | 2.75 | This study | 47.00 | [30] | U66504.1 |
|  | *Artibeus cinereus* | N/A | N/A | 2.5 | [47] | N/A | N/A | U66511.1 |
|  | *Artibeus lituratus* | N/A | N/A | 3 | [47] | N/A | N/A | HQ702537.1 |
|  | *Tonatia silvicola* | 14.15 | This study | 2.75 | This study | 32.85 | [48] | FJ155493.1 |
|  | *Trachops cirrhosus* | 16.00 ± 2.12  14.50 – 17.50 (2) | This study, [49] | 3.25 | This study | 43.87 ± 0.09  43.80 – 43.93 (2) | [30, 39] | FJ155483.1 |
|  | *Desmodus rotundus* | 15.25 | This study | 3 | This study | 28.5 | [30] | FJ155477.1 |
|  | *Diaemus youngi* | N/A | N/A | 3 | [47] | N/A | N/A | FJ155475.1 |
|  | *Anoura geoffroyi* | 7.60 | This study | 2.75 | This study | 14.1 | [30] | FJ155495.1 |
|  | *Carollia perspicillata perspicillata* | 10.13 | This study | 2.25, 2.5 | This study | 19.1 | [30] | FJ589715.1 |
|  | *Centurio senex* | 11.95 | This study | 2.75 | This study, [47] | 22 | [30] | AY604444.1 |
|  | *Glossophaga longirostris* | N/A | N/A | 2.5 | [47] | N/A | N/A | AF382874.1 |
|  | *Glossophaga soricina* | N/A | N/A | 2.5 | [47] | N/A | N/A | FJ392515.1 |
|  | *Phyllostomus discolor* | N/A | N/A | 2.5 | [47] | N/A | N/A | NA |
|  | *Phylloderma stenops* | N/A | N/A | 2.5 | [47] | N/A | N/A | FJ155480.1 |
|  | *Phyllostomus hastatus* | N/A | N/A | 3 | [47] | N/A | N/A | FJ155479.1 |
|  | *Micronycteris hirsuta* | N/A | N/A | 2.5 | [47] | N/A | N/A | DQ077415.1 |
|  | *Sturnira tildae* | N/A | N/A | 2.5 | [47] | N/A | N/A | DQ903816.1 |
|  | *Sturnira lilium* | N/A | N/A | 3 | [47] | N/A | N/A | DQ903815.1 |
|  | *Chiroderma villosum* | N/A | N/A | 3 | [47] | N/A | N/A | L28943.1 |
|  | *Vampyrops helleri* | N/A | N/A | 3 | [47] | N/A | N/A | GQ184736.1 |
| Molossidae | *Cheiromeles torquatus* | 28.53 | This study | 2.75 | This study | 135.5 | [30] | SJ. Rossiter unpub. |
|  | *Molossus molossus* | 14.07 | This study | 2.75 | This study | 16.2 | [30] | L19724.1 |
|  | *Molossus ater* | 14.6 | [36] | 3 | [31] | 37.23 ± 0.32  37.00 – 37.45 (2) | [36, 39] | HM470179.1  (*Molossus rufus*) |
|  | *Molossus major* | N/A | N/A | 3 | [31] | N/A | N/A | NA |
|  | *Molossus coebensis* | N/A | N/A | 3 | [31] | N/A | N/A | NA |
|  | *Tadarida brasiliensis* | 11.11 ± 1.55  10.01 – 12.20 (2) | This study, [50] | 2.25, 2.5 | This study, [50] | 12.5 | [30, 50] | JF489129.1 |
| Nycteridae | *Nycteris thebaica* | 9.17 | This study | 2 | This study | 11.00 | [30] | AF044653.1 |
| Natalidae | *Natalus stramineus saturates* | 9.02 | This study | 2.5 | This study | 3.9 | [30] | AY621016.1 |
|  | *Natalus tumidirostris* | N/A | N/A | 2.5 | [31] | N/A | N/A | AY621009.1 |
| Emballonuridae | *Taphozous peli* | 23.42 | This study | 3.25 | This study | 95.00 | [51] | AB444718.1  (*Saccolaimus saccolaimus*) |
|  | *Taphozous melanopogon* | 14.60 | This study | 2.25 | This study | 24.00 | HZM | EF584221.1 |
|  | *Taphozous kachensis* | 13.25 ± 1.63  12.10 – 14.40 (2) | [36, 39] | N/A | N/A | 49.20 ± 1.13  48.40 – 50.00 (2) | [36, 39] | NA |
|  | *Peropteryx macrotis* | 12.02 | This study | 2.5 | This study | 4.4 | [52] | EF584180.1 |
|  | *Saccopteryx bilineata* | 12.56 | This study | 2.5 | This study, [40] | 7.5 | [30] | EF584202.1 |
|  | *Rhynchonycteris naso* | 10.34 | This study | 2.75 | This study | 3.9 | [30] | EF584192.1 |
|  |  |  |  |  |  |  |  |  |
| Balaenidae | *Eubalaena glacialis* | 54.1 | [53] | 2.5 | [53] | 31,837,000 | [53] | DQ095151.1 |
|  | *Balaena mysticetus* | 56.5 | [53] | 2.25 | [53] | 42,201,000 | [53] | JF489130.1 |
| Balaenopteridae | *Balaenoptera musculus* | 50.6 | [53] | 2.25 | [53] | 190000000 | [43] | NC_001601.1 |
|  | *Balaenoptera acutorostrata* | 50.6 | [53] | 2.25 | [53] | 8,000,000 | [53] | NC_005271.1 |
|  | *Balaenoptera physalus* | 64.7 | [53] | 2.5 | [53] | 70000000 | [43] | NC_001321.1 |
|  | *Megaptera novaeangliae* | 60.1 | [53] | 2.0 | [53] | 30,000,000 | [53] | GQ353255.1 |
| Physeteridae | *Physeter catodon* | 54.3 | [53] | 1.75 | [53] | 20,000,000 | [53] | NC_002503.2 |
| Iniidae | *Inia geoffrensis* | 38.2 | [53] | 1.5 | [53] | 130,000 | [53] | AF334486.1 |
| Monodontidae | *Delphinapterus leucas* | 42 | [54] | N/A | N/A | 1,360,000 | [43] | U72037.1 |
| Delphinidae | *Delphinus delphis* | 30.7 | [53] | 2.25 | [53] | 70,000 | [53] | HM572302.1 |
|  | *Grampus griseus* | 41.0 | [53] | 2.5 | [53] | 387,500 | [43] | EU557095.1 |
|  | *Tursiops truncatus* | 38.9 | [53] | 2.25 | [53] | 155,000 | [53] | JN571479.1 |
|  | *Stenella attenuate* | 36.9 | [53] | 2.5 | [53] | 100,000 | [53] | *X56294.1* |
|  | *Lagenorhynchus albirostris* | 34.8 | [53] | 2.25 | [53] | 180,000 | [43] | NC_005278.1 |
|  | *Lagenorhynchus obliquidens* | 33.8 | [53] | 2.0 | [53] | 120,000 | [53] | EF093040.1 |
| Phocoenidae | *Phocoenoides dalli* | 29.1 | [53] | 2.0 | [53] | 102500 | [43] | U09678.1 |
|  | *Phocoena phocoena* | 22.5 | [53] | 1.5 | [53] | 50, 000 | [53] | U72039.1 |
| Octodontidae | *Spalacopus cyanus* | 11.68 | [55] | 3.5 | [55] | 90 | [55] | AF007061.1 |
| Spalacidae | *Spalax ehrenbergi* | 12.6 | [55] | 4 | [55] | 143 | [55] | AJ311138.1 |
| Muridae | *Pachyuromys duprasi* | 10.75 | [56] | N/A | N/A | 90 | [56] | AJ851274.1 |
|  | *Meriones unguiculatus* | 12.1 | [57] | 3.25 | [57] | 50 | [57] | AB381901.1 |
|  | *Mus musculus* | 6.0 | [55] | 1.95 | [55] | 22 | [55] | HM222709.1 |
|  | *Rattus norvegicus* | 10.7 | [55] | 2.2 | [55] | 325 | [55] | HM222710.1 |
|  | *Rattus rattus* | 12.1 | [55] | 2.125 | [55] | 200 | [55] | HM217367.1 |
| Caviidae | *Cavia porcellus* | 20.50 | [55] | 4.25 | [55] | 406 | [55] | GU136732.1 |
| Chinchillidae | *Chinchilla laniger* | 18.5 | [55] | 3.0 | [55] | 490 | [55] | AF464760.1 |
| Ctenomyidae | *Ctenomys talarum* | 10.58 | [55] | 3.25 | [55] | 140 | [55] | AF370698.1 |
| Heteromyidae | *Dipodomys merriami* | 9.83 | [56] | 3.25 | [58] | 50 | [56] | GU175438.1 |
| Cricetidae | *Arvicola terrestris* | 10.5 | [59] | 2.3 | [59] | 130 | [59] | GU954310.1 |
|  | *Microtus arvalis* | 8.5 | [59] | 2.3 | [59] | 27 | [59] | GU197794.1 |
| Bathyergidae | *Fukomys anselli* | 11.1 | [55] | 4.25 | [55] | 80 | [55] | EF043452.1  (*Fukomys mechowii*) |
| Leporidae | *Oryctolagus cuniculus* | 15.25 | [58] | 2.5 | [58] | 2,000 | [58] | HQ596486.1 |
| Cercopithecidae | *Macaca nemestrina* | 25.6 | [56] | N/A | N/A | 8,850 | [56] | EU204975.1 |
| Callitrichidae | *Callithrix (Hapale) jacchus* | N/A | N/A | 2.75 | [58] | N/A | N/A | AY434079.1 |
| Hominidae | *Homo sapiens* | 34.25 | [58] | 2.75 | [58] | 75,000 | [58] | JQ839273.1 |
| Cebidae | *Saimiri sciureus* | 15.4 | [57] | 2.25 | [57] | 743.2 | [43] | HQ005511.1 |
| Bovidae | *Bos taurus* | 38 | [56] | 3.5 | [58] | 500,000 | [56] | EU807948.1 |
|  | *Ovis aries* | N/A | N/A | 2.25 | [58] | N/A | N/A | EU365977.1 |
| Equidae | *Equus caballus* | N/A | N/A | 2.5 | [58] | N/A | N/A | JF489134.1 |
| Canidae | *Canis familiaris* | N/A | N/A | 3.25 | [58] | N/A | N/A | JF489119.1 |
| Felidae | *Felis catus* | 22.5 | [56] | 3 | [58] | 2,500 | [56] | AB194817.1 |
|  | *Panthera onca* | 33.3 | [56] | N/A | N/A | 90,000 | [56] | GU175435.1 |
|  | *Panthera tigris* | 35.5 | [56] | N/A | N/A | 106,300 | [56] | AF053028.1 |
| Otariidae | *Zalophus californianus* | 54.3 | [57] | 1.75 | [57] | 80,000 | [43] | NC_008416.1 |
| Phocidae | *Phoca vitulina* | N/A | N/A | 2.25 | [58] | N/A | N/A | AB510422.1 |
| Procyonidae | *Procyon lotor* | N/A | N/A | 2.5 | [58] | N/A | N/A | GU175439.1 |
| Elephantidae | *Elephas maximus* | 60 | [58] | 2.25 | [58] | 40,00,000 | [56] | AY769977.1 |
| Trichechidae | *Trichechus manatus* | 35 | [60] | 1.75 | [60] | 400,000 | [10] | JF489120.1 |
| Didelphidae | *Monodelphis domestica* | 6.4 | [56] | 1.8 | [61] | 110 | [56] | HQ651772.1 |
|  | *Didelphis virginiana* | 15 | [62] | 2.5 | [62, 63] | 3,000 | [64] | HM222715.1 |
| Tachyglossidae | *Tachyglossus aculeatus* | 7.61 | [65] | 0.5 | [65] | 3,500 | [65] | NC_003321.1 |
| Ornithorhynchidae | *Ornithorhynchus anatinus* | 4.43 | [65] | 0.5 | [65] | 1,400 | [65] | NC_000891.1 |

* Based on body mass of *Pteropus giganteus*

**** Out-group taxa: *Anolis poncensis* (AB377062.1) *Xenopus laevis (*NC_001573.1), *Danio rerio* (Ensembl).

Abbreviations:

NA – data missing.

**Table S3**

**Fossil calibration points used to calibrate divergence age for species phylogeny.**

| Node | Min.–Max. age:  (million years) | 5-95% Quantiles  (Mean ± S.D.) | Source: |
| --- | --- | --- | --- |
| Euarchontoglires | 61.5 – 100.5 | 61.5 - 100.7  (81.0 ± 12) | [66] |
| Carnivores | 42.8 – 63.8 | 43.13 – 62.87 (53.0 ± 6.0) | [66] |
| Human-Macaque | 23 – 33.9 | 23.57 – 33.43 (28.5 ± 3.0) | [66] |
| Marsupials | 61.7 – 71.2 | 61.15 - 71.85 (66.5 ± 3.25) | [66] |
| Ferungulata | 95.3 - 113 | 53.28 – 112.7 (104.5 ± 5.0) | [66] |
| *Craseonycteris*-Megadermatidae | 33.8 – 48.8 | 33.5 - 48.4  (41.0 ± 4.5) | [21] |
| Molossidae-Vespertilionidae-Miniopteridae | 37.1 - 56 | 37.45 – 55.55 (46.5 ± 5.5) | [21] |
| Rhinolophidae-Hipposideridae | 37.1 - 56 | 35.53 – 55.37 (46.5 ± 6.0) | [21] |
| Emballonuridae-Nycteridae | 40.2 – 58.9 | 40.45 - 58.55 (49.5 ± 5.5) | [21] |

**Table S4**

**Hearing limits (kHz) at 30 and 60 dB taken from published audiograms.**

| Species | LF (30) | LF (60) | HF (30) | HF (60) | Source of audiogram: |
| --- | --- | --- | --- | --- | --- |
| *Rousettus aegyptiacus* | 5.5 | 2.25 | 54 | 64 | [67] |
| *Rhinolophus ferrumequinum* | 9 | 4.3 | 97 | 103 | [13] |
| *Rhinopoma hardwickii* | 26.25 | - | 55 | 85 | [14] |
| *Megaderma lyra* | 6 | 70 | - | 100 | [14] |
| *Myotis lucifugus* | 12 | 10.3 | 100 | 115 | [17] |
| *Lasiurus borealis* | 10 | 5 | 55 | 72 | [68] |
| *Noctilio leporinus* | 18 | 7.5 | 64 | 111 | [18] |
| *Artibeus jamaicensis* | 7.4 | 2.8 | 80 | 130 | [69] |
| *Desmodus rotundus* | 12 | 9 | 88 | 74 | [70] |
| *Carollia perspicillata* | 9.3 | 5.2 | 126 | 150 | [71] |
| *Phyllostomus hastatus* | 4.2 | 1.77 | 87 | 105 | [72] |
| *Tadarida brasiliensis* | - | - | 70 | 100 | [39] |
| *Taphozous melanopogon* | 8 | 1.75 | 43.5 | 67.5 | [14] |
| *Taphozous kachensis* | 19 | - | 37 | 57.5 | [14] |
| *Spalacopus cyanus* | 0.28 | 0.25 | 16 | 20 | [19] |
| *Spalax ehrenbergi* | ~0.800 | 0.052 | ~0.800 | 5.9 | [73] |
| *Meriones unguiculatus* | 0.28 | - | 44 | 58 | [74] |
| *Mus musculus* | 2.3 | 3.4 | 66 | 92 | [75] |
| *Rattus norvegicus* | 0.89 | 0.53 | 64 | 68 | [76] |
| *Cavia porcellus* | 0.32 | 0.047 | 40 | 49 | [23] |
| *Chinchilla laniger* | 0.12 | 0.052 | 18.5 | 32.5 | [24] |
| *Dipodomys merriami* | 0.096 | 0.042 | 27 | 52 | [75, 77] |
| *Oryctolagus cuniculus* | 0.445 | 0.096 | 35 | 49 | [75] |
| *Macaca nemestrina* | 0.093 | - | 24.5 | 34.5 | [78] |
| *Callithrix (Hapale) jacchus* | 0.21 | - | 18 | 30 | [79] |
| *Homo sapiens* | - | 0.031 | - | 17.6 | [80] |
| *Saimiri sciureus* | 0.4 | 0.1 | 35 | 43 | [81, 82] |
| *Bos taurus* | 0.15 | 0.023 | 24 | 35 | [83] |
| *Ovis aries* | 0.44 | 0.125 | 35 | 45 | [84] |
| *Equus caballus* | 0.235 | 0.055 | 23.5 | 33.5 | [83] |
| *Canis familiaris* | 0.227 | 0.067 | 37 | 44 | [85] |
| *Felis catus* | 0.15 | 0.055 | 60 | 79 | [86] |
| *Procyon lotor* | 0.134 | - | 38 | - | [87] |
| *Elephas maximus* | 0.017 | 0.18 | 10.5 | 4.2 | [88] |
| *Zalophus californianus* | 1.4 | 0.18 | 17.5 | 33 | [89-91] |
| *Phoca vitulina* | 0.72 | 0.16 | 16.9 | 23 | [91] |
| *Didelphis viriginiana* | 2.15 | 1.03 | 45 | 68 | [92, 93] |
| *Monodelphis domestica* | 6.9 | 3.15 | 54 | 76 | [94] |

Abbreviations:

LF – low frequency hearing limit; HF - high frequency hearing limits; dB – decibel; kHZ – kilohertz.

**Table S5**

**The relationship between echolocation and relative membrane length taking phylogeny into account.**

| Model tested |  |  | Placentals |
| --- | --- | --- | --- |
|  | Parameter | All taxa | (excl. whales) |
| log basilar membrane = log body mass^0.33^ | log body mass^0.33^ | 0.37 | 0.39 |
|  | intercept | 0.75 | 0.86 |
|  | *P_MCMC_*(log body mass^0.33^) | <1x10^-4^ | <1x10^-4^ |
|  | *P_MCMC_* (intercept) | <1x10^-4^ | <1x10^-4^ |
|  | DIC | -208.90 | -158.16 |
| log basilar membrane = log body mass^0.33^ | log body mass^0.33^ | 0.41 | 0.46 |
| + laryngeal echolocation | laryngeal echolocation | -0.16 | -0.16 |
|  | intercept | 0.84 | 0.92 |
|  | *P_MCMC_*(log body mass^0.33^) | <1x10^-4^ | <1x10^-4^ |
|  | *P_MCMC_* (laryngeal echolocation) | 0.059 | 0.041 |
|  | *P_MCMC_* (intercept) | <1x10^-4^ | <1x10^-4^ |
|  | DIC | -208.05 | -157.59 |
| log basilar membrane = log body mass^0.33^ | log body mass^0.33^ | 0.38 | 0.41 |
| + CF echolocation | CF echolocation | -0.15 | -0.17 |
|  | intercept | 0.89 | 1.02 |
|  | *P_MCMC_*(log body mass^0.33^) | <1x10^-4^ | <1x10^-4^ |
|  | *P_MCMC_* (CF echolocation) | 0.016 | 0.006 |
|  | *P_MCMC_* (intercept) | <1x10^-4^ | <1x10^-4^ |
|  | DIC | -208.30 | -156.88 |

Abbreviations: M – non-bat mammals; NA – not applicable; BM^0.33^ – body mass cubed; CF – constant frequency; DIC - Deviance information criterion; *P_MCMC_ -* twice the posterior probability that a model parameter is > or < than zero (whichever is lower), as estimated by the Markov chain.

**Table S6**

**The relationship between echolocation call parameters, basilar membrane length, number of cochlear turns and body mass.**

| Model tested | Parameter | Min. freq. | Peak-energy freq. | Max. freq. |
| --- | --- | --- | --- | --- |
| log echolocation = log basilar membrane | log basilar membrane | -0.52 | -0.52 | -0.52 |
|  | intercept | 2.14 | 2.28 | 2.38 |
|  | *P_MCMC_*(log basilar membrane) | 0.011 | 0.015 | 0.010 |
|  | *P_MCMC_* (intercept) | <1 x 10^-4^ | <1 x 10^-4^ | <1 x 10^-4^ |
|  | DIC | -56.50 | -48.61 | -45.42 |
| log echolocation = log basilar membrane | log basilar membrane | -0.89 | -0.88 | -0.78 |
| + log number of turns | log number of turns | 2.25 | 2.00 | 1.44 |
|  | intercept | 1.66 | 1.88 | 2.09 |
|  | *P_MCMC_* (log basilar membrane) | <1 x 10^-4^ | <1 x 10^-4^ | 8.90 x 10^-4^ |
|  | *P_MCMC_* (log number of turns) | <1 x 10^-4^ | <5 x 10^-4^ | 0.010 |
|  | *P_MCMC_* (intercept) | <1 x 10^-4^ | <1 x 10^-4^ | <1 x 10^-4^ |
|  | DIC | -65.98 | -53.44 | -50.31 |
| log echolocation = log basilar membrane | log basilar membrane | -0.76 | -0.79 | -0.72 |
| + log number of turns + log body mass | log number of turns | 2.05 | 1.87 | 1.35 |
|  | Log body mass | -0.31 | -0.19 | -0.13 |
|  | intercept | 1.72 | 1.92 | 2.11 |
|  | *P_MCMC_* (log basilar membrane) | 8.89 x 10^-4^ | 8.90 x 10^-4^ | 0.005 |
|  | *P_MCMC_* (log number of turns) | 2.22 x 10^-4^ | 1.00 x 10^-3^ | 0.022 |
|  | *P_MCMC_* (log body mass) | 0.122 | 0.342 | 0.561 |
|  | *P_MCMC_* (intercept) | <1 x 10^-4^ | <1 x 10^-4^ | <1 x 10^-4^ |
|  | DIC | -65.54 | -54.43 | -50.98 |

Abbreviations: NA – not applicable; DIC - Deviance information criterion; *P_MCMC_ -* twice the posterior probability that a model parameter is > or < than zero (whichever is lower), as estimated by the Markov chain.

**Table S7**

**ML estimates of Pagel’s λ for inner ear parameters and body mass across the study species.**

**(A) ML estimates of λ**

| Character: | Model | lnL (k) | AIC | λ |
| --- | --- | --- | --- | --- |
| (i) Log membrane length | 1 Untransformed tree | 53.16 (3) | -100.33 | 0.899 |
|  | 2 Transformed tree (BM) | -8.05 (2) | 20.10 | 0 |
|  | 3 Transformed tree (BM) | 0.73 (2) | 2.55 | 1 |
| (ii) Log body mass^0.33^ | 1 Untransformed tree | 5.53(3) | -5.06 | 0.995 |
|  | 2 Transformed tree (BM) | -112.13 (2) | 228.27 | 0 |
|  | 3 Transformed tree (BM) | 4.99 (2) | -5.99 | 1 |
| (iii) Log spirals | 1 Untransformed tree | 144.81 (3) | -283.63 | 0.963 |
|  | 2 Transformed tree (BM) | 82.56 (2) | -161.13 | 0 |
|  | 3 Transformed tree (BM) | 136.31 (2) | -268.61 | 1 |

**(B) LRTs of estimated λ**

| Character: | Model comparisons: | LRT | *P* (1 d.f.) |
| --- | --- | --- | --- |
| (i) Log membrane length | 2 vs. 1 | 122.42 | 1.87 x 10^-28^ |
|  | 3 vs. 1 | 104.88 | 1.30 x 10^-24^ |
| (ii) Log body mass^0.33^ | 2 vs. 1 | 235.33 | 4.09 x 10^-53^ |
|  | 3 vs. 1 | 1.08 | 0.30 |
| (iii) Log spirals | 2 vs. 1 | 124.50 | 6.55 x 10^-29^ |
|  | 3 vs. 1 | 17.01 | 3.71 x 10^-05^ |

ML estimates of Pagel’s λ for log relative and absolute basilar membrane length, log body mass and log number of cochlear turns across the taxa included in the study using the calibrated species tree and transformed trees.

Abbreviations: AIC - Akaike information criterion; ML – maximum likelihood; BM – Brownian motion; lnL – log likelihood; LRT – likelihood ration test; λ - Pagel’s lamda;.

**Table S8**

**Averaged echolocation call frequencies used by this study, taken from published sources.**

| Species name | Peak-energy freq. | Min. freq. | Max. freq. | Sources |
| --- | --- | --- | --- | --- |
| *Rousettus aegyptiacus* | 35.03 | 12.00 | 70.00 | [95] and sources within. |
| *Rhinolophus ferrumequinum* | 81.17 | 66.98 | 80.04 | [96]; Sources cited within [97]; [98]; [40]; Sources cited within [99]; [33]; [96]; [100]; [101]; [102]; [103]. |
| *Rhinolophus rouxi* | 73.36 | 60.17 | 73.17 | [104]; [101]; [105]; [106]. |
| *Rhinolophus philippinensis (Medium)* | 41.70 | 41.70 | 41.70 | Recorded in the field. |
| *Rhinolophus philippinensis (Small)* | 53.70 | 53.70 | 53.70 | Recorded in the field. |
| *Rhinolophus philippinensis (Large)* | 27.00 | 27.00 | 27.00 | Recorded in the field. |
| *Rhinolophus megaphyllus* | 69.17 | 67.00 | 71.00 | [107]; [108]; [102]. |
| *Rhinolophus pearsonii* | 61.88 | 56.20 | 70.00 | [33]; [101]; [109]. |
| *Rhinolophus marshalli* | 40.40 | 39.20 | 50.55 | www.bristol.ac.uk/biology/research/behaviour/batlab/ |
| *Rhinolophus affinis* | 78.28 | 70.00 | 88.50 | [33]; [109]; [28]; [101]; [110]. |
| *Hipposideros ridleyi* | 63.45 | 61.90 | 65.00 | [28]; [110]. |
| *Cloeotis percivali* | 212.00 | 183.00 | 212.00 | [111]. |
| *Hipposideros fulvus* | 157.00 | 157.49 | 157.61 | [112]. |
| *Hipposideros gigas* | 62.20 | 50.00 | 66.00 | [113]; Sources cited within [97]; [111]; Sources cited within [99]; [114]. |
| *Hipposideros speoris* | 136.25 | 132.00 | 139.00 | [112]; [106]. |
| *Hipposideros bicolor* | 134.18 | 130.90 | 142.00 | [28]; [115]. |
| *Rhinopoma microphyllum* | 28.00 | 27.00 | 30.00 | [116]. |
| *Rhinopoma hardwickii* | 34.88 | 32.00 | 40.00 | [117]; [118]; [116]; [102]. |
| *Macroderma gigas* | 45.00 | 40.00 | 50.00 | [102]. |
| *Cardioderma cor* | 56.70 | 39.60 | 90.90 | [119]. |
| *Megaderma spasma* | 19.50 | 17.00 | 22.00 | Sources cited within [97]. |
| *Megaderma lyra* | 8.00 | 30.00 | 47.52 | [120]; [39]; |
| *Lavia frons* | 18.50 | 16.90 | 82.90 | [119]. |
| *Craseonycteris thonglongyai* | 73.00 | 73.00 | 73.00 | [121]. |
| *Myotis lucifugus* | 75.43 | 39.14 | 44.88 | [122] and sources within; Sources cited within [97]; [111]; [102]. |
| *Myotis muricola* | 57.20 | 53.70 | 79.90 | [123]. |
| *Miniopterus schreibersii* | 56.07 | 52.10 | 85.20 | [102]. |
| *Scotophilus kuhlii* | 43.30 | 36.60 | 84.90 | [123]. |
| *Murina suilla* | 85.00 | 55.20 | 165.00 | [124]. |
| *Murina cyclotis* | 77.20 | 51.60 | 165.20 | [124]. |
| *Scotomanes ornatus* | 31 | 22.85 | 73.89 | [125]. |
| *Lasiurus borealis* | 37.50 | 30.00 | 45.00 | [68]. |
| *Plecotus auritus* | 43.20 | 32.60 | 52.00 | [102]; [126]; |
| *Pipistrellus pipistrellus* | 105.30 | 44.60 | 120.00 | [127]; [128]; [31]; [129]; [102]. |
| *Eptesicus serotinus* | 32.30 | 26.35 | 58.70 | [127]; Sources cited within [97]. |
| *Nyctalus noctula* | NA | 22.50 | 25.00 | [98]. |
| *Noctilio leporinus* | 53.57 | 30.13 | 61.00 | Sources cited within [97]; [31]; [102]; Sources cited within [130]. |
| *Pteronotus macleayi grisea* | 75.00 | 70.00 | 80.00 | [45]. |
| *Pteronotus parnellii* | 60.36 | 48.00 | 64.00 | [102]; Sources cited within [97]; [131]; [47]. |
| *Pteronotus davyi* | 73.05 | 60.50 | 68.00 | [131]; [132]; Sources cited within [97]. |
| *Mormoops megaphylla* | 42.50 | 37.00 | 48.00 | [47]. |
| *Artibeus jamaicensis* | 67.15 | 54.00 | 91.20 | [47]; Sources cited within [97]; [133]; [134]. |
| *Tonatia saurophila (T. silvicola)* | 56.50 | 51.50 | 71.00 | [135]. |
| *Trachops cirrhosus* | 79.00 | 53.00 | 79.00 | [136]. |
| *Desmodus rotundus* | 72.98 | 49.85 | 82.10 | [130]; [135]; [47]; [39]; Sources cited within [97]. |
| *Anoura geoffroyi* | 102.00 | 51.00 | 112.00 | [47]. |
| *Carollia perspicillata perspicillata* | 82.80 | 70.00 | 125.00 | [137]; [47]; [102]; [71]; Sources cited within [97]. |
| *Centurio senex* | 115.00 | 70.00 | 115.00 | [47]. |
| *Cheiromeles torquatus* | 38.60 | 18.63 | 26.57 | [138]. |
| *Molossus molossus* | 44.67 | 22.70 | 50.30 | [102]; [139]. |
| *Molossus ater* | 42.00 | 27.50 | 42.50 | [139]. |
| *Tadarida brasiliensis* | 27.07 | 22.48 | 33.29 | [102]; |
| *Nycteris thebaica* | 88.23 | 55.97 | 75.50 | Sources cited within [97]; [111]; [114]; |
| *Natalus stramineus saturates* | 42.95 | 33.00 | 70.00 | [134]. |
| *Natalus stramineus saturates* | 113.80 | 79.80 | 152.80 | [134]. |
| *Peropteryx macrotis* | 40.00 | 20.00 | 45.00 | [140]. |
| *Taphozous melanopogon* | 26.95 | 25.20 | 28.70 | [102]; [123]. |
| *Saccopteryx bilineata* | 42.56 | 41.20 | 45.80 | Sources cited within [97]; [141]; [131]; [40]; [140]; [102]; [142]. |
| *Rhynchonycteris naso* | 98.20 | 64.00 | 99.83 | [143]; [140]; [135]. |
| *Thyroptera tricolor* | 45.00 | 25.00 | 45.00 | [143]. |

**Table S9**

**Published frequency of maximum energy (ie. CF component of call) and morphological parameters for Rhinolophidae species.**

| Species name | Call Freq.  (kHz) | Body mass  (g) | Fore arm  (mm) | Ref. |
| --- | --- | --- | --- | --- |
| *Rhinolophus acuminatus* | 89.0 | - | 48.0 | [28] |
| *Rhinolophus affinis* | 77.6 | 13.8 | 49.3 | [110] |
| *Rhinolophus arcuatus* | 66.5 | - | 42.0-53.5 | Sources cited within [144], [8] |
| *Rhinolophus beddomei* | 49.3 | 16.0 | 53.0 | [145] |
| *Rhinolophus blasii* | 86.6 | 10.0 | 48.0 | [146] |
| *Rhinolophus borneensis* | 81.8 | - | 43.0 | [28] |
| *Rhinolophus capensis* | 81.9-85.5 | 9.5–16.0 | 47-52 | [147] & sources cited within |
| *Rhinolophus clivosus* | 92.1 | 18.0 | 54 | [146] |
| *Rhinolophus coelophyllus* | 80.88 | - | 40-46 | [148], [8] |
| *Rhinolophus cornutus* | 105.1 | 4.37 | 36.99 | [101] |
| *Rhinolophus creaghi* | 68.0 | - | 50.0 | [28] |
| *Rhinolophus darlingi* | 88.1 | 9.1 | 47 | [146] |
| *Rhinolophus deckeni* | 72.0 | 15.5 | 49.9 | [149] |
| *Rhinolophus denti* | 110 | 6.0 | 42 | [111], [150] |
| *Rhinolophus euryale* | 104.0 | - | 42.5-51.0 | [144], [8] |
| *Rhinolophus ferrumequinum* | 81.0 | 22.6 | 53.0-62.5 | [144], [30], [8] |
| *Rhinolophus ferrumequinum nippon* | 75.1 | 15.32 | 58.8 | [101] |
| *Rhinolophus formosae* | 43-45 | - | 57-61 | [151] |
| *Rhinolophus fumigatus* | 53.8 | 12.3 | 52 | [146] |
| *Rhinolophus hildebrandti* | 33.0 | 29.3 | 67 | [146] |
| *Rhinolophus hipposideros* | 110.0 | - | 34-42 | [144], [8] |
| *Rhinolophus landeri* | 107.3 | 7.5 | 44 | [146] |
| *Rhinolophus lepidus* | 98.0 | - | 41 | [28] |
| *Rhinolophus luctus* | 42.0 | 26.3 | 63.4 | [110] |
| *Rhinolophus macrotis* | 48.0 | 7.5 | 45.5 | [110] |
| *Rhinolophus malayanus* | 78 | - | 41 | [28] |
| *Rhinolophus marshalli* | 41.8–44.5 | 5.8–7.0 | 38.9–47.1 | [33] |
| *Rhinolophus megaphyllus* | 67-71 | 7-14 | 44.0-51.0 | [152], [153] |
| *Rhinolophus mehelyi* | 109.0 | - | 47.0-53.0 | [144], [8] |
| *Rhinolophus microglobosus (male)* | 96.2 | 6.1 | 44.0 | [154] |
| *Rhinolophus monoceros (male)* | 111.77 | 4.8 | 37.02 | [155] |
| *Rhinolophus osgoodi* | 95.2 | 6.9 | 45.4 | [156] |
| *Rhinolophus paradoxolophus* | 43.0 | 10.8 | 55.2 | [157] |
| *Rhinolophus parcus* | 107.0 | 3.5 | 37.0 | [101] |
| *Rhinolophus pearsoni* | 56.2 | 17.43 | 55.93 | [101] |
| *Rhinolophus philippinensis* | 36.6 | - | 50.0 | [28] |
| *Rhinolophus philippinensis (small)* | 53.70 | 6.5 | 47.4 | SJ Rossiter, T Kingston |
| *Rhinolophus philippinensis (medium)* | 41.70 | 7.0 | 48.4 | SJ Rossiter, T Kingston |
| *Rhinolophus philippinensis (large)* | 27.00 | 12.0 | 55.3 | SJ Rossiter, T Kingston |
| *Rhinolophus pumilus (male)* | 105.6-119.3 | - | 36.1-42.1 | [158] |
| *Rhinolophus cf. pusilius* | 100.0 | - | 35 | [28] |
| *Rhinolophus refulgens* | 99.7 | 6.3 | 40.5 | [110] |
| *Rhinolophus rex* | 23.7–26.4 | 10.8–14 | 51.6–57.3 | [33] |
| *Rhinolophus robinsoni* | 67.0 | - | 44.0 | [28] |
| *Rhinolophus rouxii* | 72.8 | 12.8 | 50.5 | [101] |
| *Rhinolophus sedulus* | 66.8 | 7.7 | 40.3 | [110] |
| *Rhinolophus shameli* | 76.0 | - | 44.0 | [28] |
| *Rhinolophus siamensis* | 60.0–69.3 | 5.5–6.4 | 39.2–45.4 | [33] |
| *Rhinolophus simulator* | 84.1 | 7.1 | 44 | [159] |
| *Rhinolophus sinicus* | 78.9–88.5 | 8.5–14.3 | 44.1–54.4 | [33] |
| *Rhinolophus stheno* | 86.1 | 8.9 | 48.8 | [110] |
| *Rhinolophus swinnyi* | 107 | 7.8 | 44 | [146] |
| *Rhinolophus thomasi* | 80.0 | 10.13 | 44.96 | [101] |
| *Rhinolophus trifoliatus* | 53.3 | 13.9 | 50.9 | [110] |
| *Rhinolophus yunanensis* | 50.95 | - | 51.50-64 | [148], [8] |

**Supplementary References:**

1. Welker KL, Orkin JD, Ryan TM: **Analysis of intraindividual and intraspecific variation in semicircular canal dimensions using high-resolution x-ray computed tomography.** *J Anat* 2009, **215:**444-451.

2. Miller JD: **Sex diffferences in the length of the organ of Corti in humans.** *J Acoust Soc Am* 2007, **121:**EL151-EL155.

3. Kingston T, Rossiter SJ: **Harmonic-hopping in Wallacea's bats.** *Nature* 2004, **429:**654-657.

4. Hadfield JD: **MCMC methods for multi-response generalized linear mixed models: The MCMCglmm R Package.** *J Stat Softw* 2010, **33:**1-22.

5. Hadfield JD, Nakagawa S: **General quantitative genetic methods for comparative biology: phylogenies, taxonomies and multi-trait models for continuous and categorical characters.** *J Evol Biol* 2010, **23:**494-508.

6. Larkin MA, Blackshields G, Brown NP, Chenna R, McGettigan PA, McWilliam H, Valentin F, Wallace IM, Wilm A, Lopez R, et al: **Clustal W and Clustal X version 2.0.** *Bioinformatics* 2007, **23:**2947-2948.

7. Drummond AJ, Rambaut A: **BEAST: Bayesian evolutionary analysis by sampling trees.** *BMC Evol Biol* 2007, **7:**214.

8. Csorba G, Ujhelyi P, Thomas N: *Horseshoe bats of the world (Chiroptera: Rhinolophidae).* Shropshire, United Kingdom: Alana Books; 2003.

9. Dannhof BJ, Bruns V: **The organ of Corti in the bat *Hipposideros bicolor*.** *Hear Res* 1991, **53:**253-268.

10. Gerstein ER, Gerstein L, Forsythe SE, Blue JE: **The underwater audiogram of the West Indian manatee (*Trichechus manatus*).** *J Acoust Soc Am* 1999, **105:**3575-3583.

11. Lewis-Oritt N, Porter CA, Baker RJ: **Molecular systematics of the family Mormoopidae (Chiroptera) based on cytochrome b and recombination activating gene 2 sequences.** *Mol Phylogenet Evol* 2001, **20:**426-436.

12. Harmon LJ, Weir JT, Brock CD, Glor RE, Challenger W: **GEIGER: investigating evolutionary radiations.** *Bioinformatics* 2008, **24:**129-131.

13. Long GR, Schnitzler H-U: **Behavioral audiograms from the bat, *Rhinolophus ferrumequinum*.** *J Comp Physiol*, **100:**211-219.

14. Neuweiler G, Singh S, Sripathi K: **Audiograms of a South Indian bat community.** *J Comp Physiol A* 1984, **154:**133-142.

15. Thabah A, Li G, Wang YN, Liang B, Hu KL, Zhang SY, Jones G: **Diet, echolocation calls, and phylogenetic affinities of the great evening bat (*Ia io;* Vespertilionidae): Another carnivorous bat.** *J Mammal* 2007, **88:**728-735.

16. McGowen MR, Spaulding M, Gatesy J: **Divergence date estimation and a comprehensive molecular tree of extant cetaceans.** *Mol Phylogenet Evol* 2009, **53:**891-906.

17. Dalland JI: **Hearing sensitivity in bats.** *Science* 1965, **150:**1185-1186.

18. Wenstrup JJ: **Auditory sensitivity in the fish-catching bat, *Noctilio leporinus*.** *J Comp Physiol A* 1984, **155:**91-101.

19. Begall S, Burda H, Schneider B: **Hearing in Coruros (*Spalacopus cyanus*): special audiogram features of a subterranean rodent.** *J Comp Physiol A* 2004, **190:**963-969.

20. Zhou X, Xu S, Xu J, Chen B, Zhou K, Yang G: **Phylogenomic analysis resolves the interordinal relationships and rapid diversification of the Laurasiatherian mammals.** *Syst Biol* 2012, **61:**150-164.

21. Meredith RW, Janecka JE, Gatesy J, Ryder OA, Fisher CA, Teeling EC, Goodbla A, Eizirik E, Simao TLL, Stadler T, et al: **Impacts of the Cretaceous Terrestrial Revolution and KPg Extinction on Mammal Diversification.** *Science* 2011, **334:**521-524.

22. Drummond AJ, Ho SYW, Phillips MJ, Rambaut A: **Relaxed phylogenetics and dating with confidence.** *PLoS Biol* 2006, **4:**699-710.

23. Heffner R, Heffner H, Masterton R.B.: **Behavioral measurement of absolute and frequency-difference thresholds in guinea pig.** *J Acoust Soc Am* 1971, **49:**1888-1895.

24. Heffner RS, Heffner HE: **Behavioral hearing range of the chinchilla.** *Hear Res* 1991, **52:**13-16.

25. Posada D: **jModelTest: Phylogenetic model averaging.** *Mol Biol Evol* 2008, **25:**1253-1256.

26. Tamura K, Peterson D, Peterson N, Stecher G, Nei M, Kumar S: **MEGA5: Molecular evolutionary genetics analysis using Maximum Likelihood, Evolutionary Distance, and Maximum Parsimony Methods.** *Mol Biol Evol* 2011, **28:**2731-2739.

27. Jones KE, Bininda-Emonds ORP, Gittleman JL: **Bats, clocks, and rocks: Diversification patterns in chiroptera.** *Evolution* 2005, **59:**2243-2255.

28. Francis CM, Habersetzer J: **Interspecific and intraspecific variation in echolocation call frequency and morphology of horseshoe bats, *Rhinolophus* and *Hipposideros*.** In *Bat Biology and Conservation* Edited by Kunz TH, Racey P. Washington and London: Smithsonian Institution Press; 1998

29. Cox PG, Jeffery N: **Semicircular canals and agility: the influence of size and shape measures.** *J Anat* 2010, **216:**37-47.

30. Norberg UM, Rayner JMV: **Ecological morphology and flight in bats (Mammalia; Chiroptera): wing adaptations, flight performance, foraging strategy and echolocation.** *Phil Trans R Soc Lond B* 1987, **316:**335-427.

31. Pye A: **The megachiroptera and vespertilionoidea of the microchiroptera.** *J Morphol* 1966, **119:**101-119.

32. Echteler S, Fay R, Popper A: **Structure of the mammalian cochlea.** In *Comparative hearing: mammals.* Edited by Fay R, Popper A. New York: Springer-Verlag; 1994: 134-171

33. Zhang LB, Jones G, Zhang JS, Zhu GJ, Parsons S, Rossiter SJ, Zhang SY: **Recent surveys of bats (Mammalia: Chiroptera) from China. I. Rhinolophidae and Hipposideridae.** *Acta Chiropt* 2009, **11:**71-88.

34. Melzer P: **A deoxyglucose study on auditory responses in the bat *Rhinolophus rouxi*.** *Brain Res Bull* 1985, **15:**677-681.

35. Struebig MJ: *Bat diversity and ecology in lowland forest, southern Borneo. Final report of the UEA Kalimantan Bat Expedition (2004)* 2005.

36. Kossl M, Vater M: **Cochlear structure and function in bats.** In *Hearing by bats.* Edited by Popper A, Fay R. New York: Springer-Verlag; 1995: 191-234

37. Smith AT, Xie Y (Eds.): **A guide to the mammals of China.** Princeton: Princeton University Press; 2008.

38. Barclay RMR, Brigham RM: **Prey detection, dietary niche breadth, and body size in bats - why are aerial insectivorous bats so small?** *Am Nat* 1991, **137:**693-703.

39. Neuweiler G: *The Biology of Bats.* New York and Oxford: Oxford University Press; 2000.

40. Pye A: **The structure of the cochlea in chiroptera. I. Microchiroptera: Emballonuroidea and Rhinolophoidea.** *J Morphol* 1966, **118:**495-510.

41. Csada R: ***Cardioderma cor*.** *Mamm Species* 1996, **519:**1-4.

42. Borisenko A, Kruskop S: *Bats of Vietnam and adjacent territories. An identification manual.* Geos; 2003.

43. Smith F, Lyons S, Ernest S, Jones K, Kaufman D, Dayan T, Marquet P, Brown J, Haskell J: **Body mass of late Quaternary mammals.** *Ecology* 2003, **84:**3403.

44. Spoor F, Garland T, Krovitz G, Ryan TM, Silcox MT, Walker A: **The primate semicircular canal system and locomotion.** *Proc Natl Acad Sci U S A* 2007, **104:**10808-10812.

45. Mancina CA: ***Pteronotus macleayii*.** *Mamm Species* 2005, **778:**1-3.

46. Rydell J, Arita HT, Santos M, Granados J: **Acoustic identification of insectivorous bats (order Chiroptera) of Yucatan, Mexico.** *J Zool* 2002, **257:**27-36.

47. Pye A: **The structure of the cochlea in chiroptera. III. Microchiroptera: Phyllostomatoidea.** *J Morphol* 1967, **121:**241-254.

48. Mendellin RA, Arita HT: ***Tonatia evotis* and *Tonatia silvicola*.** *Mamm Species* 1989, **334:**1-5.

49. Bruns V, Burda H, Ryan MJ: **Ear morphology of the frog-eating (*Trachops cirrhosus*, Family, Phyllostomidae) - Apparent specializations for low-frequency hearing.** *J Morphol* 1989, **199:**103-118.

50. Vater M, Siefer W: **The cochlea of *Tadarida brasiliensis*: Specialized functional organization in a generalized bat.** *Hear Res* 1995, **91:**178-195.

51. Norberg UM: **Allometry of bat wings and legs and comparison with bird wings.** *Phil Trans R Soc Lond B* 1981, **292:**359-398.

52. Yee D: ***Peropteryx macrotis*** *Mamm Species* 2000, **643:**1-4.

53. Ketten DR: **Cetacean Ears.** In *Hearing by Whales and Dolphins.* Edited by Au WWL, Popper AN, Fay RR. New York: Springer; 2000: 43-108.[Fay RR, popper AN (Series Editor): *Springer Handbook of Auditory Research* ].

54. Ketten DR: **Structure and function in whale ears.** *Bioacoustics* 1997, **8:**103-135.

55. Begall S, Burda H: **Acoustic communication and burrow acoustics are reflected in the ear morphology of the coruro (*Spalacopus cyanus*, Octodontidae), a social fossorial rodent.** *J Morphol* 2006, **267:**382-390.

56. Kirk EC, Gosselin-Ildari AD: **Cochlear labyrinth volume and hearing abilities in primates.** *Anat Rec* 2009, **292:**765-776.

57. Manoussaki D, Chadwick RS, Ketten DR, Arruda J, Dimitriadis EK, O'Malley JT: **The influence of cochlear shape on low-frequency hearing.** *Proc Natl Acad Sci U S A* 2008, **105:**6162-6166.

58. West CD: **The relationship of the spiral turns of the cochlea and the length of the basilar membrane to the range of audible frequencies in ground dwelling mammals.** *J Acoust Soc Am* 1985, **77:**1091-1101.

59. Lange S, Stalleicken J, Burda H: **Functional morphology of the ear in fossorial rodents, *Microtus arvalis* and *Arvicola terrestris*.** *J Morphol* 2004, **262:**770-779.

60. Ketten DR, Odell DK, Domning DP: **Structure, function, and adaptation of the manatee ear.** In *Marine Mammal Sensory Systems.* Edited by Thomas JA, Kastelein RA, Supin AY. New York: Plenum Press; 1992: 77-95

61. Ekdale EG: **Ontogenetic variation in the bony labyrinth of *Monodelphis domestica* (Mammalia: Marsupialia) following ossification of the inner ear cavities.** *Anat Rec* 2010, **293:**1896-1912.

62. Fernandez C, Schmidt RS: **Opossum ear and evolution of coiled cochlea.** *J Comp Neurol* 1963, **121:**151-159.

63. McCrady E: **The embryology of the opossum.** *Amer Anat Mem* 1938, **16:**1-233.

64. de Magalhaes JP, Costa J: **A database of vertebrate longevity records and their relation to other life-history traits.** *J Evol Biol* 2009, **22:**1770-1774.

65. Ladhams A, Pickles JO: **Morphology of the monotreme organ of Corti and macula lagena.** *J Comp Neurol* 1996, **366:**335-347.

66. Benton MJ, Donoghue PCJ: **Paleontological evidence to date the tree of life.** *Mol Biol Evol* 2007, **24:**26-53.

67. Koay G, Heffner RS, Heffner HE: **Hearing in a megachiropteran fruit bat (*Rousettus aegyptiacus*).** *J Comp Psychol* 1998, **112:**371-382.

68. Obrist MK, Wenstrup JJ: **Hearing and hunting in red bats (*Lasiurus borealis*, Vespertilionidae): Audiogram and ear properties.** *J Exp Biol* 1998, **201:**143-154.

69. Heffner RS, Koay G, Heffner HE: **Hearing in American leaf-nosed bats. III: *Artibeus jamaicensis*.** *Hear Res* 2003, **184:**113-122.

70. Schmidt U, Schlegel P, Schweizer H, Neuweiler G: **Audition in vampire bats, *Desmodus rotundus*.** *J Comp Physiol A* 1991, **168:**45-51.

71. Koay G, Heffner RS, Bitter KS, Heffner HE: **Hearing in American leaf-nosed bats. II: *Carollia perspicillata*.** *Hear Res* 2003, **178:**27-34.

72. Koay G, Bitter KS, Heffner HE, Heffner RS: **Hearing in American leaf-nosed bats. 1: *Phyllostomus hastatus*.** *Hear Res* 2002, **171:**96-102.

73. Heffner RS, Heffner HE: **Hearing and sound localization in blind mole rats (*Spalax ehrenbergi*).** *Hear Res* 1992, **62:**206-216.

74. Ryan A: **Hearing sensitivity of the Mongolian gerbil, *Meriones unguiculatis*.** *J Acoust Soc Am* 1976, **59:**1222-1226.

75. Heffner H, Masterton R: **Hearing in glires: domestic rabbit, cotton rat, feral house mouse and kangaroo rat.** *J Acoust Soc Am* 1980, **68:**1584-1599.

76. Heffner HE, Heffner RS, Contos C, Ott T: **Audiogram of the hooded Norway rat.** *Hear Res* 1994, **73:**244-248.

77. Webster DB, Webster M: **Kangaroo rat auditory thresholds before and after middle ear reduction.** *Brain Behav Evol* 1972, **5:**41-53.

78. Stebbins WC, Green S, Miller FL: **Auditory sensitivity of the monkey.** *Science* 1966, **153:**1646-1647.

79. Seiden HR: **Auditory acuity of the marmoset monkey (*Hapale jacchus*).** Princeton University, 1958.

80. Jackson LL, Heffner RS, Heffner HE: **Free-field audiogram of the Japanese macaque (*Macaca fuscata*).** *J Acoust Soc Am* 1999, **106:**3017-3023.

81. Beecher M: **Pure tone thresholds of the squirrel monkey (*Saimiri sciureus*).** *J Acoust Soc Am* 1974, **55:**196-198.

82. Green S: **Auditory sensitivity and equal loudness in the squirrel monkey (*Saimiri sciureus*).** *J Exp Anal Behav* 1975, **23:**255-264.

83. Heffner R, Heffner H: **Hearing in large mammals: The horse (*Equus caballus*) and cattle (*Bos taurus*).** *Behav Neurosci* 1983, **97:**299-309.

84. Wollack CH: **The auditory acuity of the sheep (*Ovis aries*).** *J Aud Res* 1963, **3:**121-132.

85. Heffner HE: **Hearing in large and small dogs: Absolute thresholds and size of the tympanic membrane.** *Behav Neurosci* 1983, **97:**310-318.

86. Heffner RS, Heffner HE: **Hearing range of the domestic cat.** *Hear Res* 1985, **19:**85-88.

87. Wollack CH: **Auditory thresholds in the raccoon (*Procyon lotor*).** *J Aud Res* 1965, **5:**139-144.

88. Heffner RS, Heffner HE: **Hearing in the elephant (*Elephas maximus*) - absolute sensitivity, frequency, frequency discrimination, and sound localization.** *J Comp Physiol Psychol* 1982, **96:**926-944.

89. Schusterman RJ: **Auditory sensitivity ofthe California sea lion to airborne sound.** *J Acoust Soc Am* 1974, **56:**1248-1251.

90. Schusterman RJ: **Behavioral capabilitiesof seals and sea lions: A review of their hearing, visual, learning and diving skills.** *Psychol Rec* 1981, **31:**125-143.

91. Kastak D, Schusterman RJ: **Low-frequency amphibious hearing in pinnipeds: Methods, measurements, noise,and ecology.** *J Acoust Soc Am* 1998, **103:**2216-2228.

92. Ravizza RJ, Heffner HE, Masterton B: **Hearing in primitive mammals: I, Opossum (*Didelphis virginiana*).** *J Aud Res* 1969, **9:**1-7.

93. Ravizza RJ, Masterton B: **Contribution of neocortex to sound localization in opossum (*Didelphis virginiana*).** *J Neurophysiol* 1972, **35:**344-356.

94. Frost SB, Masterton RB: **Hearing in primitive mammals: *Monodelphis domestica* and *Marmosa elegans*.** *Hear Res* 1994, **76:**67-72.

95. Holland RA, Waters DA, Rayner JMV: **Echolocation signal structure in the Megachiropteran bat *Rousettus aegyptiacus* Geoffroy 1810.** *J Exp Biol* 2004, **207:**4361-4369.

96. Jones G, Rayner JMV: **Foraging behavior and echolocation of wild horseshoe bats *Rhinolophus ferrumequinum* and *R. hipposideros* (Chiroptera, Rhinolophidae).** *Behav Ecol Sociobiol* 1989, **25:**183-191.

97. Goudy-Trainor A, Freeman PW: **Call parameters and facial features in bats: a surprising failure of form following function.** *Acta Chiropt* 2002, **4:**1-16.

98. Vogler B, Neuweiler G: **Echolocation in the noctule (*Nyctalus noctula*) and horseshoe bat (*Rhinolophus ferrumequinum*).** *J Comp Physiol* 1983, **152:**421-432.

99. Bogdanowicz W, Fenton MB, Daleszczyk K: **The relationships between echolocation calls, morphology and diet in insectivorous bats.** *J Zool* 1999, **247:**381-393.

100. Taniguchi I: **Echolocation sounds and hearing of the greater Japanese horseshoe bat (*Rhinolophus ferrumequinum nippon*).** *J Comp Physiol A* 1985, **156:**185-188.

101. Zhao HH, Zhang SY, Zuo MX, Zhou J: **Correlations between call frequency and ear length in bats belonging to the families Rhinolophidae and Hipposideridae.** *J Zool* 2003, **259:**189-195.

102. Neuweiler G: **Auditory adaptations for prey capture in echolocating bats.** *Physiol Rev* 1990, **70:**615-641.

103. Parsons S, Jones G: **Acoustic identification of 12 species of echolocating bat by discriminant function analysis and artificial neural networks.** *J Exp Biol* 2000, **203:**2641-2656.

104. Neuweiler G, Metzner W, Heilmann U, Rubsamen R, Eckrich M, Costa HH: **Foraging behavior and echolocation in the rufous horseshoe bat (*Rhinolophus rouxi*) of Sri Lanka.** *Behav Ecol Sociobiol* 1987, **20:**53-67.

105. Rubsamen R: **Ontogenesis of the echolocation system in the rufous horseshoe bat, *Rhinolophus rouxi* (audition and vocalization in early postnatal development).** *J Comp Physiol A* 1987, **161:**899-913.

106. Schuller G: **Hearing characteristics and doppler shift compensation in south Indian CF-FM bats.** *J Comp Physiol* 1980, **139:**349-356.

107. Pavey CR, Burwell CJ: **Bat predation on eared moths: a test of the allotonic frequency hypothesis.** *Oikos* 1998, **81:**143-151.

108. Jones G, Corben C: **Echolocation calls from six species of microchiropteran bats in south-eastern Queensland.** *Aust Mammal* 1993, **16:**35-38.

109. Robinson MF: **A relationship between echolocation calls and noseleaf widths in bats of the genera *Rhinolophus* and *Hipposideros*.** *J Zool* 1996, **239:**389-393.

110. Kingston T, Jones G, Zubaid A, Kunz TH: **Resource partitioning in rhinolophoid bats revisited.** *Oecologia* 2000, **124:**332-342.

111. Fenton MB, Bell GP: **Recognition of species of insectivorous bats by their echolocation calls.** *J Mammal* 1981, **62:**233-243.

112. Jones G, Sripathi K, Waters DA, Marimuthu G: **Individual variation in the echolocation calls of three sympatric Indian hipposiderid bats, and an experimental attempt to jam bat echolocation.** *Folia Zool* 1994, **43:**347 - 362.

113. Pye A: **Structure of Cochlea in Chiroptera from Africa.** *Period Biol* 1973, **75:**83-87.

114. Aldridge HDJN, Rautenbach IL: **Morphology, echolocation and resource partitioning in insectivorous bats.** *J Anim Ecol* 1987, **56:**763-778.

115. Kingston T, Lara MC, Jones G, Akbar Z, Kunz TH, Schneider CJ: **Acoustic divergence in two cryptic Hipposideros species: a role for social selection?** *Proc R Soc B* 2001, **268:**1381-1386.

116. Davis L: **An introduction to the bats of the United Arab Emirates.** *ECHOES Ecology Ltd, Marchmont Avenue, Polmont, Scotland* 2007**:**available at http://www.echoesecology.co.uk/documents/BatsoftheUAE_000.pdf.

117. Habersetzer J: **Adaptive echolocation sounds in the bat *Rhinopoma hardwickei* a field study.** *J Comp Physiol* 1981, **144:**559-566.

118. Simmons JA, Kick SA, Lawrence BD: **Echolocation and hearing in the mouse-tailed bat, *Rhinopoma hardwickei:* acoustic evolution of echolocation in bats.** *J Comp Physiol A* 1984, **154:**347-356.

119. Taylor PJ, Geiselman C, Kabochi P, Agwanda B, Turner S: **Intraspecific variation in the calls of some African bats (Order Chiroptera).** *Durban Mus Novit* 2005, **30:**24-37.

120. Schmidt S, Hanke S, Pillat J: **The role of echolocation in the hunting of terrestrial prey - new evidence for an underestimated strategy in the gleaning bat, *Megaderma lyra*.** *J Comp Physiol A* 2000, **186:**975-988.

121. Surlykke A, Miller LA, Mohl B, Andersen BB, Christensendalsgaard J, Jorgensen MB: **Echolocation in two very small bats from Thailand - *Craseonycteris thonglongyai* and *Myotis siligorensis*.** *Behav Ecol Sociobiol* 1993, **33:**1-12.

122. Thomas DW, Bell GP, Fenton MB: **Variation in echolocation call frequencies recorded from North American Vespertilionid bats: a cautionary note.** *J Mammal* 1987, **68:**842-847.

123. Pottie SA, Lane DJW, Kingston T, Lee BPY-H: **The microchiropteran bat fauna of Singapore.** *Acta Chiropt* 2005, **7:**237-247.

124. Kingston T, Jones G, Akbar Z, Kunz TH: **Echolocation signal design in Kerivoulinae and Murininae (Chiroptera: Vespertilionidae) from Malaysia.** *J Zool* 1999, **249:**359-374.

125. Liu H, Shi H-y, Fan Q-P, Chen W-y, Li B-b: **Echolocation Calls of Scotomanes ornatus.** *J Mianyang Normal Univ* 2001, **30:**69-71.

126. Russo D, Jones G: **Identification of twenty-two bat species (Mammalia: Chiroptera) from Italy by analysis of time-expanded recordings of echolocation calls.** *J Zool* 2002, **258:**91-103.

127. Miller LA, Degn HJ: **The acoustic behavior of four species of vespertilionid bats studied in the field.** *J Comp Physiol* 1981, **142:**67-74.

128. Waters DA, Jones G: **Echolocation call structure and intensity in 5 species of insectivorous bats.** *J Exp Biol* 1995, **198:**475-489.

129. Surlykke A, Miller LA: **The influence of arctiid moth clicks on bat echolocation; jamming or warning ?** *J Comp Physiot A* 1985, **156:**831-843.

130. Bohn KM, Moss CF, Wilkinson GS: **Correlated evolution between hearing sensitivity and social calls in bats.** *Biol Lett* 2006, **2:**561-564.

131. O'Farrell MJ, Miller BW: **A new examination of echolocation calls of some Neotropical bats (Emballonuridae and Mormoopidae) using Anabat.** *J Mammal* 1997, **78:**954-963.

132. Ibanez C, Guillen A, Juste B. J, Perez-Jorda J: **Echolocation calls of *Pteronotus davyi* (Chiroptera: Mormoopidae) from Panama.** *J Mammal* 1999, **80:**924-928.

133. Brinklov S, Kalko EKV, Surlykke A: **Intense echolocation calls from two 'whispering' bats, *Artibeus jamaicensis* and *Macrophyllum macrophyllum* (Phyllostomidae).** *J Exp Biol* 2009, **212:**11-20.

134. Jennings NV, Parsons S, Barlow KE, Gannon MR: **Echolocation calls and wing morphology of bats from the West Indies.** *Acta Chiropt* 2004, **6:**75-90.

135. Pio DV, Clarke FM, Mackie I, Racey PA: **Echolocation calls of the bats of Trinidad, West Indies: is guild membership reflected in echolocation signal design?** *Acta Chiropt* 2010, **12:**217-229.

136. Barclay RMR, Fenton MB, Tuttle MD, Ryan MJ: **Echolocation calls produced by *Trachops cirrhosus* while hunting frogs.** *Can J Zool* 1981, **59:**750-753.

137. Sterbing SJ: **Postnatal development of vocalizations and hearing in the Phyllostomid bat, *Carollia perspicillata*.** *J Mammal* 2002, **83:**516-525.

138. Kingston T, Jones G, Akbar Z, H. KT: **Alternation of echolocation calls in 5 species of aerial-feeding insectivouros bats from Malaysia.** *J Mammal* 2003, **84:**205-215.

139. Vater M, Schlegel P, Zoller H: **Comparative auditory neurophysiology of the inferior colliculus of two molossid bats, *Molossus ater* and *Molossus molossus.* I. Gross evoked potentials and single unit responses to pure tones.** *J Comp Physiol* 1979, **131:**137-145.

140. Jung K, Kalko EKV, Von Helversen O: **Echolocation calls in Central American emballonurid bats: signal design and call frequency alternation.** *J Zool* 2007, **272:**125-137.

141. Barclay RMR: **Echolocation calls of emballonurid bats from Panama.** *J Comp Physiol* 1983, **151:**515-520.

142. Kalko EKV: **Echolocation signal design, foraging habitats and guild structure in six neotropical sheath-tailed bats (Emballonuridae).** *Symp Zool Soc Lond* 1995, **67:**259-273.

143. Fenton MB, Rydell J, Vonhof MJ, Eklöf J, Lancaster WC: **Constant frequency and frequency-modulated components in the echolocation calls of three species of small bats (Embalonuridae, Thyropteridae, and Vespertilionidae).** *Can J Zool* 1999, **77:**1891-1900.

144. Heller KG, von Helversen O: **Resource partitioning of sonar frequency bands in rhinolophoid bats.** *Oecologia* 1989, **80:**178-186.

145. Soisook P, Niyomwan P, Srikrachang M, Srithongchuaya T, Bates PJJ: **Discovery of *Rhinolophus beddomei* (Chiroptera: Rhinolophidae) from Thailand with a brief comparison to other related taxa.** *Trop Nat Hist* 2010, **10:**67-79.

146. Jacobs DS, Barclay RMR, Walker MH: **The allometry of echolocation call frequencies of insectivorous bats: why do some species deviate from the pattern?** *Oecologia* 2007, **152:**583-594.

147. Stoffberg S: ***Rhinolophus capensis* (Chiroptera: Rhinolophidae).** *Mamm Species* 2008, **810:**1-4.

148. Hughes AC, Satasook C, Bates PJJ, Soisook P, Sritongchuay T, Jones G, Bumrungsrind S: **Echolocation call analysis and presence-only modelling as conservation monitoring tools for Rhinolophoid bats in Thailand.** *Acta Chiropt* 2010, **12:**311-327.

149. Monadjem A, Schoeman MC, Reside A, Pio DV, Stoffberg S, Bayliss J, Cotterill FPDW, Curran M, Kopp M, Taylor PJ: **A recent inventory of the bats of Mozambique with documentation of seven new species for the country.** *Acta Chiropt* 2010, **12:**371-391.

150. Stuart C, Stuart T: *A field guide to mammals of Southern Africa* Cape Town: Stuik Publishers; 2001.

151. Lin LK, Lee LL, Cheng HC: *Bats of Taiwan.* 2nd edn: Taichung, Taiwan: National Museum of Natural Science; 2004.

152. Pavey CR, Burwell CJ: **Cohabitation and predation by insectivorous bats on eared moths in subterranean roosts.** *J Zool* 2005, **265:**141-146.

153. Pavey CR, Burwell CJ: **Bat predation on eared moths: a test of the allotonic frequency hypothesis.** *Oikos* 1998, **81:**143-151.

154. Soisook P, Bumrungsri S, Satasook C, Thong VD, Bu SSH, Harrison DL, Bates PJJ: **A taxonomic review of *Rhinolophus stheno* and *R. malayanus* (Chiroptera: Rhinolophidae) from continental Southeast Asia: an evaluation of echolocation call frequency in discriminating between cryptic species.** *Acta Chiropt* 2008, **10:**221-242.

155. Chen SF, Jones G, Rossiter SJ: **Determinants of echolocation call frequency variation in the Formosan lesser horseshoe bat (*Rhinolophus monoceros*).** *Proc R Soc B* 2009, **276:**3901-3909.

156. **http://www.bio.bris.ac.uk/research/bats**

157. Eger JL, Fenton MB: ***Rhinolophus paradoxolophus*.** *Mamm Species* 2003, **731:**1-4.

158. Yoshino H, Matsumura S, Kinjo K, Tamura H, Ota H, Izawa M: **Geographical variation in echolocation call and body size of the Okinawan least horseshoe bat, *Rhinolophus pumilus* (Mammalia: Rhinolophidae), on Okinawa-jima island, Ryukyu archipelago, Japan.** *Zoolog Sci* 2006, **23:**661-667.

159. Monadjem A, Reside A, Lumsden L: **Echolocation calls of rhinolophid and hipposiderid bats in Swaziland.** *S Afr J Wildl Res* 2007, **37:**9-15.
