# Supplementary figures and images for: Evolutionary origins of ultrasonic hearing and laryngeal echolocation in bats inferred from morphological analyses of the inner ear
Source: Front Zool. 2013 Jan 30;10:2. doi: 10.1186/1742-9994-10-2 (PMC3598973; doi:10.1186/1742-9994-10-2)

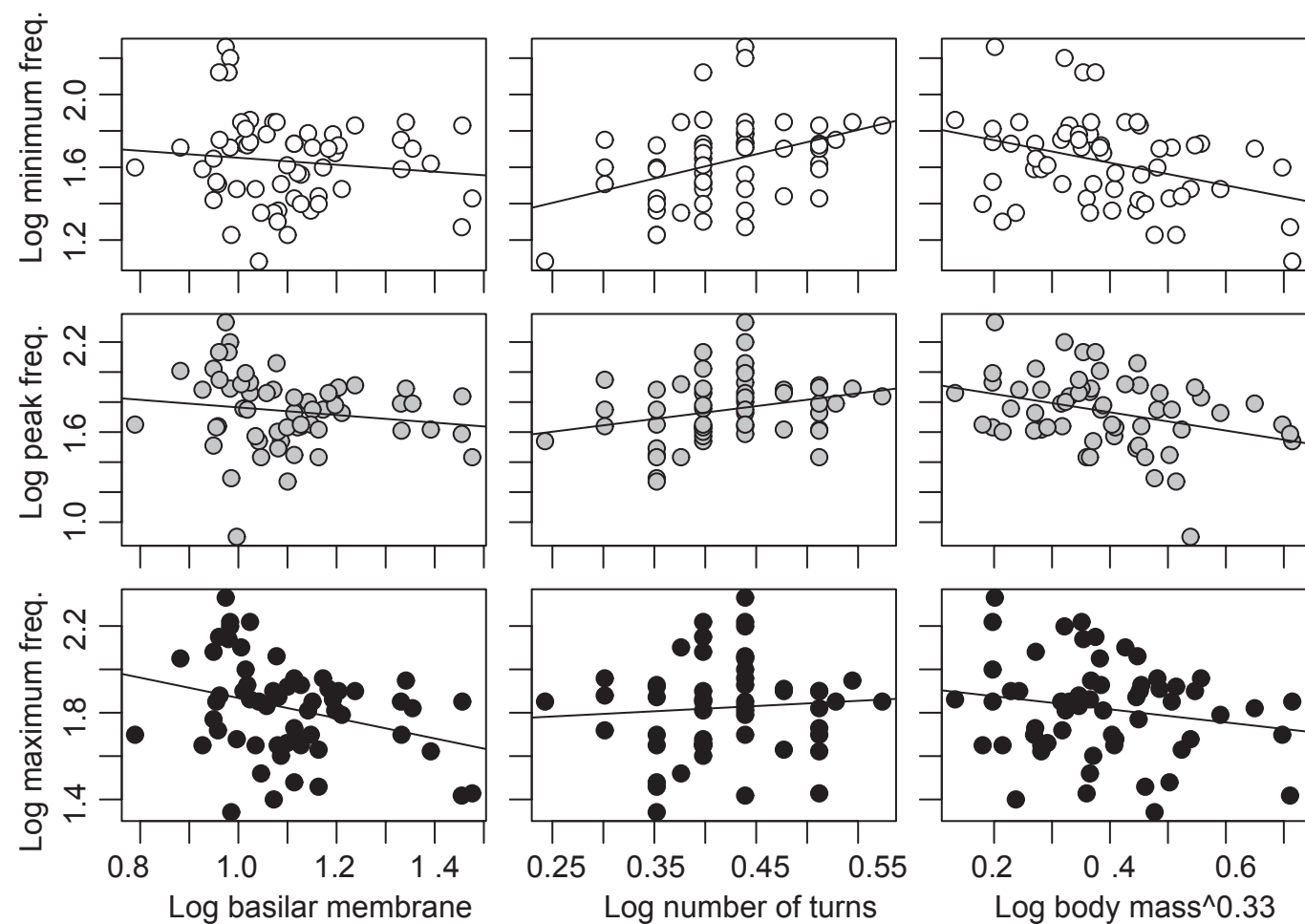

Supplement: Additional file 2: Figure S2 — Multiple regression plots of echolocation call parameters, basilar membrane length, number of cochlear turns and body mass. Stepwise multiple regressions suggest that equations with only inner ear parameters were the best fitting models: log maximum frequency = -0.96 log basilar membrane + 1.58 log turns + 2.22, multiple R2= 0.26, F = 9.01 (2, 51 d.f.), P = 4 x 10^-4^; log peak energy frequency = -1.01 log basilar membrane + 2.26 log turns + 1.92, multiple R2= 0.36, F = 14.05 (2, 51 d.f.), P = 1.38 x 10^-5^; log minimum frequency = -1.02 log basilar membrane + 2.76 log turns + 1.60, multiple R2= 0.40, F = 17.09 (2, 51 d.f.), P = 2.09 x 10^-6^. [file 1742-9994-10-2-S2.pdf]

(A)

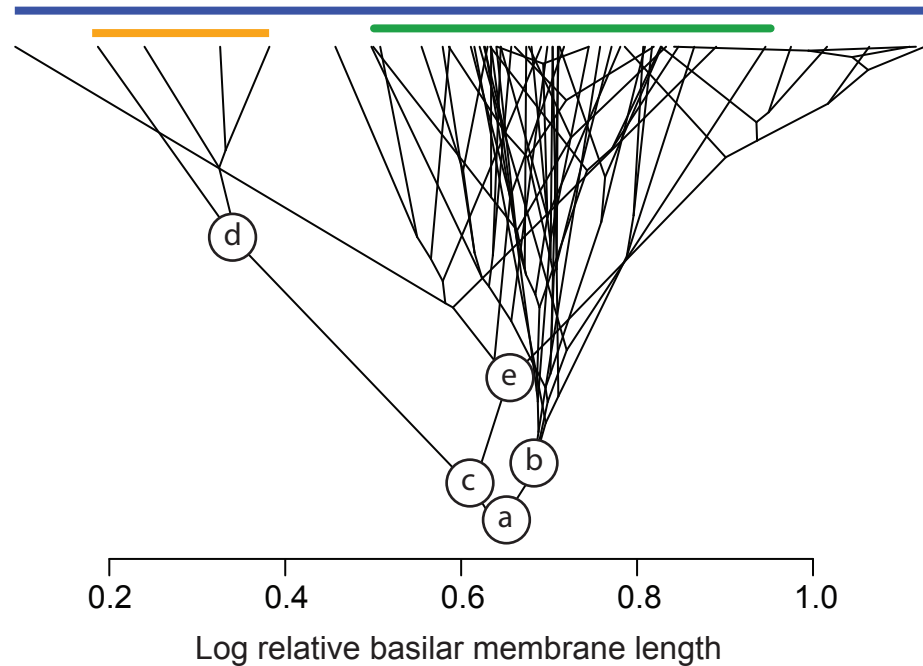

(B)

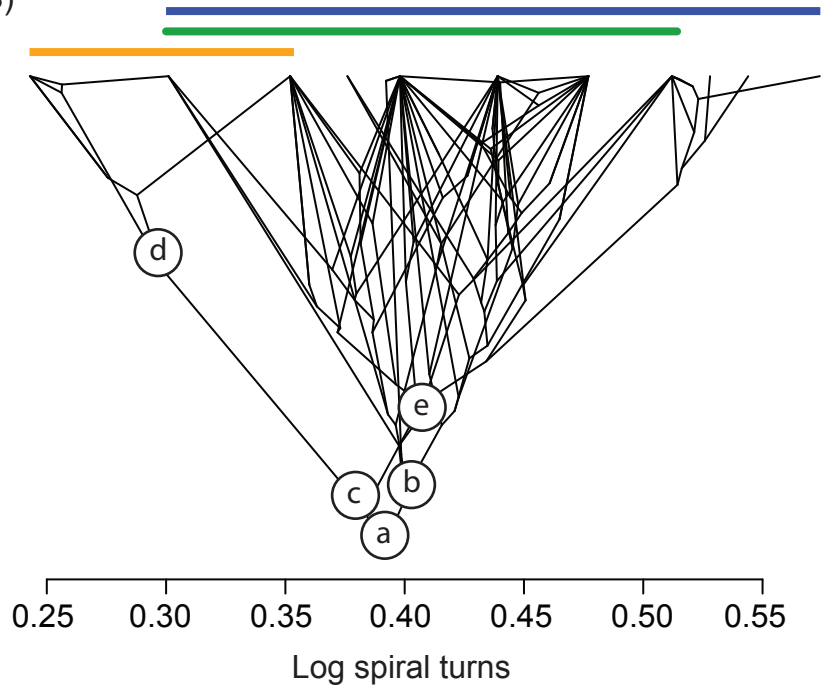

Supplement: Additional file 3: Figure S3 — Maximum likelihood ancestral reconstructions of bat inner ears - (A) relative basilar membrane length and (B) number of cochlear turns. Phylogenies and character values are depicted as ‘Traitgrams’, whereby the position along the y-axis corresponds to node age in millions of years and position along the x-axis corresponds to the reconstructed character value. Coloured bars indicate key subdivisions within bats: Old World fruit bats (orange); echolocating Yinpterochiroptera (blue); Yangochiroptera (green). Keys nodes: bat common ancestor (a); Yangochiroptera common ancestor (b); Yinpterochiroptera common ancestor (c); Old World fruit bat common ancestor (d); echolocating Yinpterochiroptera common ancestor (e). [file 1742-9994-10-2-S3.pdf]

(A)

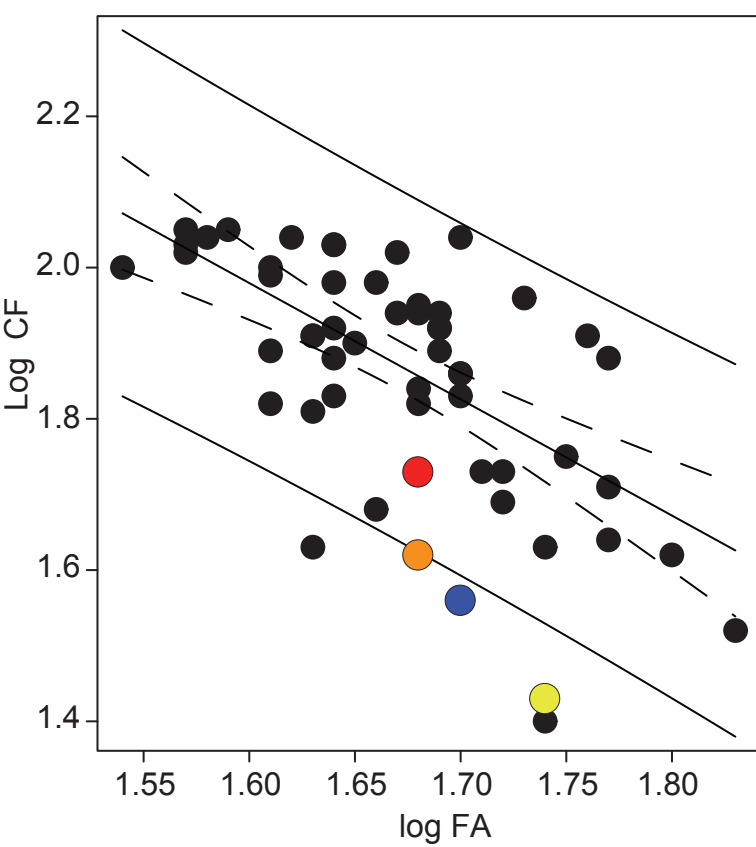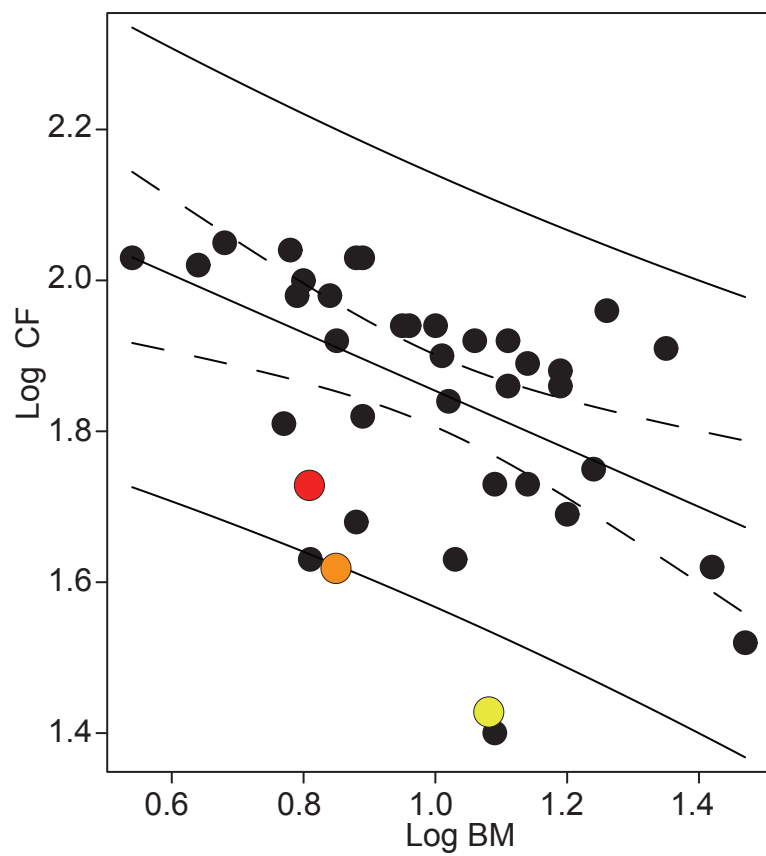

(B)

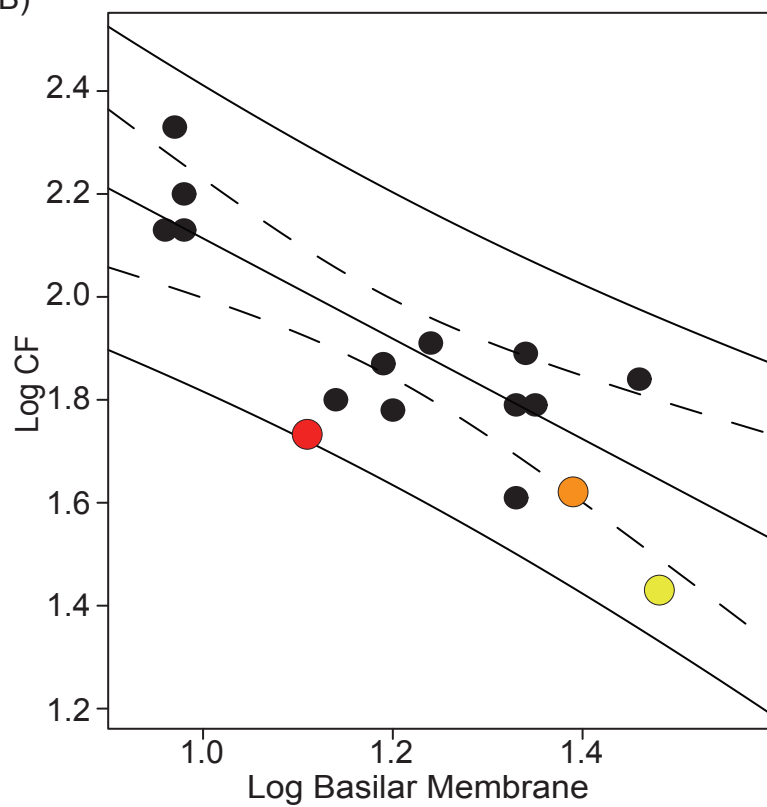

Supplement: Additional file 4: Figure S4 — Morphological parameters versus echolocation call frequency in Rhinolophus species. Published values for species taken from literature (black points), the three Rhinolophus philippinensis size morphs measured by this study: small (red), medium (orange), large (yellow) and values published for one R. philippinensis values taken from [28] (blue). (A) Average forearm length, body mass and echolocation call frequency for Rhinolophus spp. from values obtained from literature sources. A significant negative relationship was found (log CF = -1.54 log forearm + 4.44; R2 = 0.42, F = 36.57, P = 1.98 x 10^-7^ and log CF = -0.38 log body mass + 2.24; R2 = 0.25, F = 12.34, P = 0.001). (B) The relationship between basilar membrane length and constant frequency echolocation call (log CF = -0.98 log basilar membrane + 3.09; R2 = 0.63, F = 21.74, P < 0.001). [file 1742-9994-10-2-S4.pdf]

(A)

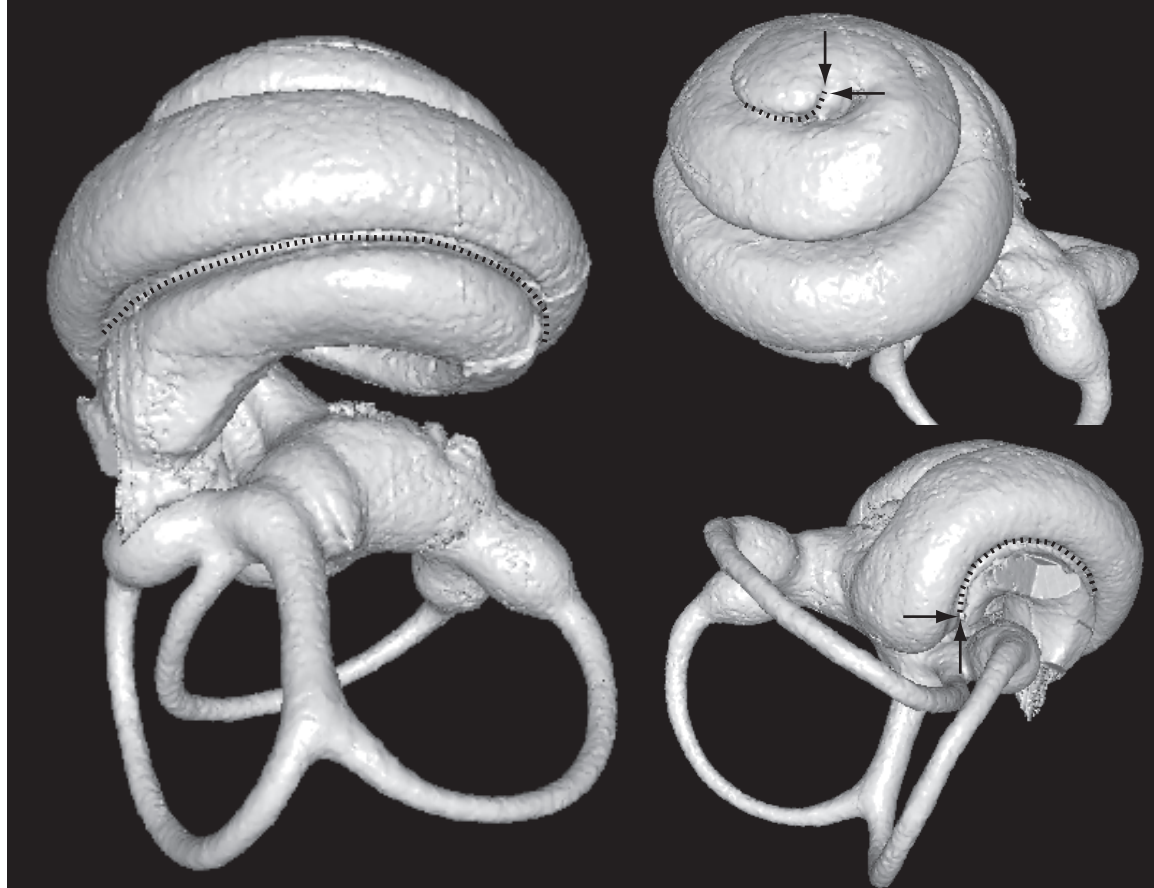

(B)

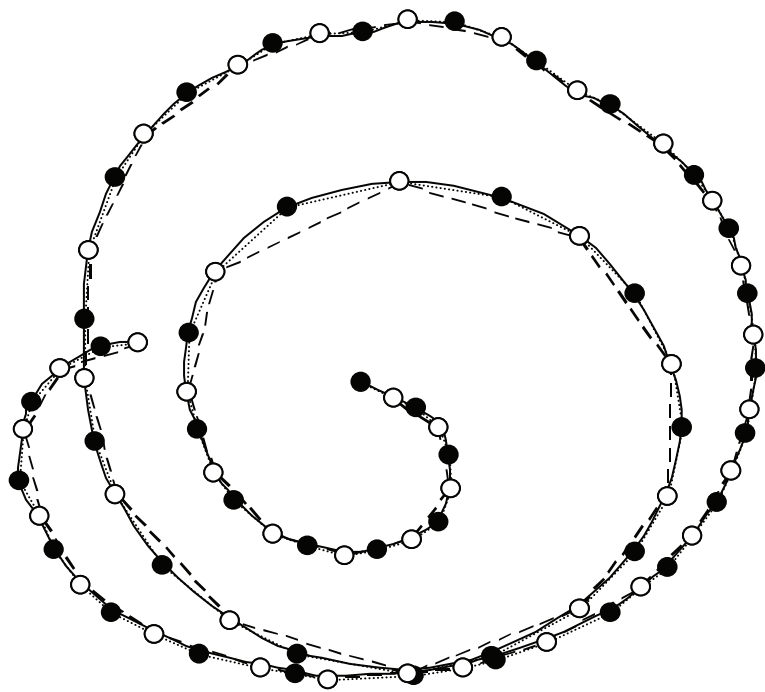

Supplement: Additional file 5: Figure S1 — Measuring basilar membrane length from reconstructed inner ear endocasts. (A) Left: Medial view of the right cochlear endocast of Craseonycteris thonglongyai (specimen number HZM.1.34982, ref. Table S1). A representation of the path of the basilar membrane measured by this study is shown by the dotted line. Right upper: Apical view of the right cochlear endocast of C. thonglongyai (HZM.1.34982). Black arrows correspond to the end point of the representation of the path of the basilar membrane (dotted line) measured by this study. Right lower: Medial view of the right cochlear endocast of C. thonglongyai (HZM.1.34982). Black arrows correspond to the start point of the representation of the path of the basilar membrane (dotted line) measured by this study. (B) Two-dimensional plots showing the representative paths of the basilar membrane for the right cochlea of Pipistrellus pipistrellus, using either 86 or 43 landmark points, connected with straight connecting lines. The basilar membrane path as depicted by a smoothed curvilinear path is also superimposed over these points. The estimated length calculated from the subset of 43 coordinates was only 8.304 mm, compared to 8.473 mm from 86 coordinates. This corresponds to a negative difference of 0.170 mm or a 2% underestimate of membrane length. Furthermore, the path traced by the straight lines connecting the 86 points much more faithfully follows that of the curved path. Therefore, 86 landmark points were deemed to be a suitable compromise between efficiency and accuracy and was used to collect all basilar membrane estimates. The 86 landmarks used in this study (circles); curved path between points (black line); straight lines between points used to estimate basilar membrane length (dotted line); 43 points (white circles), and the dashed line the straight line distance between white circles (dashed line). [file 1742-9994-10-2-S5.pdf]
